# On the relevance of serial cyclone clustering for Arctic sea ice

**DOI:** 10.1038/s41467-026-76245-5

**Published:** 2026-08-05

**Authors:** Lars Aue, Sofie Tiedeck, Peter Finocchio, Timo Vihma, Petteri Uotila, Gunnar Spreen, Annette Rinke

**Affiliations:** 1 Alfred Wegener Institute, Helmholtz Center for Polar and Marine Research, Potsdam, Germany; 2 Naval Research Laboratory (NRL), Monterey, California USA; 3 Finnish Meteorological Institute, Helsinki, Finland; 4 Institute for Atmospheric and Earth System Research/Physics, Faculty of Science, University of Helsinki, Helsinki, Finland; 5 University of Bremen, Institute of Environmental Physics, Bremen, Germany

**Keywords:** Atmospheric science, Cryospheric science

## Abstract

Short-term changes in Arctic sea-ice concentration (SIC) are largely driven by weather, and strong ice loss typically occurs during intense Arctic cyclones causing unusually warm and stormy conditions. Such conditions are prolonged when several cyclones follow rapidly on each other. Here, we analyze changes in SIC for such periods of serial cyclone clustering utilizing satellite observations and reanalysis data from 1979-2024. While cyclones generally decrease SIC compared to non-cyclone conditions, cyclone clusters impact the ice approximately 2.5 times longer and cause two times stronger overall SIC-reductions than solitary cyclones. The amount of SIC-loss due to cyclone clusters scales with the intensity and number of clustered cyclones, and intensified SIC-loss occurs from 2004-2024 compared to 1979-1999, even within fixed SIC-categories. These findings highlight the relevance of accumulated impacts of clustered weather events for Arctic sea-ice variability and emphasize the need to understand drivers of serial cyclone clustering in the Arctic.

## Abstract (web summary)

Cyclone tracking and satellite sea-ice observations reveal the importance of cyclone clustering in the Arctic: Clusters of cyclones cause much greater sea-ice loss than isolated cyclones, and their impacts have intensified over recent decades.

## Introduction

The Arctic sea ice is an important habitat^1^ and climate component^2–4^. Its rapidly ongoing decline marks a fundamental transition in the Arctic Ocean^5^, with drastic consequences within the Arctic itself, and potentially significant yet uncertain impacts in the mid-latitudes^6–8^. Predicting the timing and extent of Arctic sea-ice decline is thus of high societal relevance. A prerequisite for this task is to identify and understand the key processes impacting Arctic sea ice. From an atmospheric perspective, these impacts on sea ice are strongly shaped by episodic weather events, such as cyclones^9^.

Strong cyclones can trigger extreme and very rapid sea-ice loss events, as exemplified by the Great Arctic Cyclone in August 2012. This storm strongly contributed to the low Arctic sea-ice extent observed in September 2012 by fracturing large portions of the ice pack and triggering a fourfold rise in basal melt due to upward mixing of relatively warm ocean water^10–12^. Such examples from the recent past are complemented by model studies suggesting that the occurrence of strong, individual cyclones can be the final push to reach the first ice-free day in the Arctic^13^, a major milestone in future Arctic climate change. Furthermore, the effects of individual cyclones can accumulate and modulate seasonal-scale climate interactions, including the atmospheric control over ocean heat transport into the Arctic^14^. From a more practical perspective, strong ice drift and enhanced wave activity during cyclone-related storms can also affect maritime economic activities in the Arctic^15,16^, which have expanded considerably in recent years due to the increased accessibility and shorter shipping routes^17,18^. At the same time, Arctic coastal communities are increasingly exposed to hazardous weather events due to intensified cyclone activity^19,20^, while reductions in sea-ice cover and thawing permafrost have increased the vulnerability of coastal environments to storm-related impacts^21^.

Previous studies revealed that the impact of cyclones on sea ice is rather complex^22^: It depends on the considered time (days before/during vs. after cyclone), on the cyclone characteristics (intensity, path), and the traversed sea-ice conditions (particularly ice concentration). Subsequently, the impact varies across cyclone events, regions, and seasons.

In the cold season, cyclones advect heat and moisture into the Arctic, enhance downward longwave radiation and stall or occasionally reverse sea-ice growth^23–25^. Cyclonic precipitation building up snow cover atop the sea ice is another mechanism to hinder sea-ice growth, especially for thinner ice^26,27^. However, extensive precipitation on top of thin ice may result in snow-ice formation, increasing the ice thickness. With thinning sea ice, these processes may become more common in the Arctic^28^. Dynamical processes such as ice drift, divergence and deformation are as important for sea-ice reduction during cyclone passages as thermodynamic processes^29–32^. Additionally, wave-sea ice interactions triggered by cyclone-induced large swells can impact sea-ice cover and thickness in winter directly via ice break-up and enhanced lateral ice melt^33^ as well as indirectly by washing away the snow pack on top of sea ice^34^.

In the warm season, Arctic cyclones have a weaker average intensity than cyclones in other seasons^35^, but can still have significant local impacts on sea ice. Cyclone winds locally reduce sea-ice concentration (SIC) by enhancing sea-ice divergence, poleward ice drift, and warm-air advection over sea ice^35,36^. Cyclone winds also locally enhance the basal melt of sea ice by mixing warmer seawater upward from below the shallow summer mixed layer^11,37,38^. Unlike in the cold season, the enhanced cloud cover associated with warm-season cyclones reduces the downward shortwave radiative flux at the surface, which can locally slow down the seasonal loss of sea ice^29,39,40^. These opposing effects of warm-season cyclones result in a comparatively small overall impact on SIC^22,29^. However, the thinning ice pack and warming of the upper ocean over the last two decades have contributed to a shift toward greater ice loss caused by cyclones at the end of the melt season^41^.

In previous studies, Arctic cyclones have been considered as separate events affecting the sea ice independently of each other. In contrast, strong accumulated impacts during phases of serial cyclone clustering have already been identified for other regions on Earth and other target quantities such as wind damage, precipitation, or sea level extremes^42,43^. Serial cyclone clustering also occurs in the Arctic^44^, but attempts to link this phenomenon to sea-ice changes are so far limited to case studies of specific cyclone events^31^ or of specific sea-ice (loss) events, such as autumnal pauses in arctic-wide sea-ice expansion^45^. In particular, no systematic analysis of the effects of serial cyclone clusters on sea ice, involving a large number of cases, has yet been conducted.

To address this knowledge gap, we statistically quantify the impact of serial clusters of multiple Arctic cyclones (in the following short “cyclone clusters" or “cyclone clustering") on SIC and compare it to the impact of solitary cyclones in terms of magnitude and duration based on ERA5 reanalysis data^46^. Our analysis covers the cold season (NDJFM) and the warm season (MJJA), as well as two different time periods (2004–2024 vs 1979–1999) in order to identify recent changes in cyclone cluster impacts on SIC. We further evaluate how the properties of cyclone clusters and background sea-ice conditions modulate the impact on SIC.

## Results

### Cyclone clustering in the Arctic

First, we present a cyclone cluster climatology for 2004–2024 based on ERA5 reanalysis data^46^ and a cyclone tracking tool^47^, which we also compare to the earlier period 1979–1999. To detect cyclone clusters, we apply an absolute threshold requiring a minimum of two consecutive cyclones with a maximum time gap of 48 h between them. This approach offers flexibility in terms of the number of cyclones per cluster and the total cluster duration. Lowering the time threshold from 48h to 36 h reduces the number of detected clusters, particularly outside the Arctic along the cold-season storm tracks, but does not qualitatively alter the results (Supplementary Fig. 1).

**Fig. 1:**
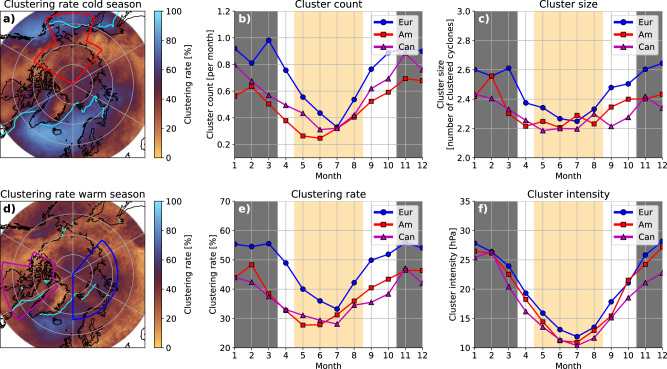
Occurrence and properties of cyclone clusters in the Arctic. Panels **a**, **d** indicate clustering rate (share of all cyclones which occur within clusters) for the cold season (NDJFM, (**a**)) and warm season (MJJA, (**d**)) for the years 2004–2024, and the light blue lines indicate the average position of sea-ice edge (15% mean SIC contour). The remaining panels indicate the annual cycle of absolute cyclone cluster count (**b**), clustering rate (**e**), cluster size (**c**) and cluster intensity (**f**) for the Eurasian Arctic ("Eur", blue domain in panel (**d**)), the Amerasian Arctic ("Am", red domain in panel (**a**)) and the Canadian Archipelago sector ("Can", purple domain in panel (**d**)). Gray and yellow shadings highlight months in the cold and warm seasons, respectively.

Within the Arctic, there are pronounced regional and seasonal variations in cyclone cluster occurrence (Fig. 1a, d). In alignment with previous studies^42^, we find that cyclone clustering occurs frequently in the exit regions of the North Atlantic storm track in the cold season. These relatively high clustering rates of more than 70% extend further into the Atlantic marginal seas of the Arctic Ocean. Similarly high values are found in the Pacific sector of the Arctic, including the Gulf of Alaska, the Bering Sea and the Sea of Okhotsk. In the central Arctic Ocean, the cold season clustering rate is around 50%, and in the Beaufort, Chukchi and East Siberian Seas, most cyclones occur separately from each other. In the warm season, clustering rates are substantially lower along the Atlantic and Pacific storm tracks compared to the cold season. As a consequence, the regional maximum of Arctic warm-season clustering rate is found in the Kara Sea and the central Arctic Ocean and amounts to approximately 50%. Throughout basically the entire study domain, seasonal clustering rates did not change significantly between the periods 1979–1999 and 2004–2024 (Supplementary Fig. 2).

Next, we divide the sea-ice-covered regions of the Arctic into subdomains and analyze the annual cycle of cluster occurrence and properties. We define an Eurasian and an Amerasian sector of the Arctic Ocean following a previous study^41^, but in this study extend the Amerasian Arctic southward to include the cold season ice edge located in the Bering Sea. We further introduce an additional Canadian Archipelago sector. All three domains are outlined in Fig. 1.

The occurrence frequency of cyclone clusters exhibits a clear minimum in July for the Eurasian Arctic in both absolute counts and clustering rate (Fig. 1b, e). In contrast, in the Amerasian Arctic, the minimum cyclone cluster occurrence is already reached in May to June. Hereby, the magnitude of the annual cycle is larger in the Eurasian Arctic than in the Amerasian Arctic and the Canadian Archipelago. This also applies to the mean cluster size (Fig. 1c), defined as the number of cyclones per cluster. Despite the differences in terms of cluster occurrence, the annual cycle of cluster intensity (mean intensity of all clustered cyclones) is very similar for all three regions (Fig. 1f), except for a slightly lower intensity in the Canadian Archipelago from October through December. As a consequence, the annual cycles of cluster occurrence and cluster intensity are shifted in time in the Amerasian Arctic (occurrence minimum in May-June, intensity minimum in June-July). While monthly cluster occurrence and properties are similar for 2004–2024 and 1979–1999 in the Eurasian Arctic, some deviations are found for the Amerasian Arctic, including a more pronounced yearly minimum in the clustering rate (and cluster size) in June for 1979–1999 than for the more recent period (Supplementary Fig. 2).

### Amplification of sea-ice impact due to cyclone clustering

Next, we evaluate changes in SIC from one day before to the last day of each cyclone (cluster) passage (denoted “during") for all ice-covered grid-cells within the cyclone (cluster) area, focusing on the years 2004–2024. From those changes, we subtract a non-cyclone reference calculated for the same number of days and at the same grid-cells, but for days not impacted by cyclones. Details are found in the Methods section.

**Fig. 2:**
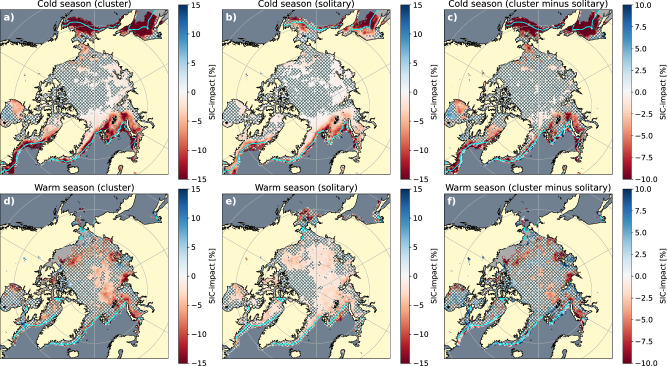
Impact of cyclone clusters and solitary cyclones on sea ice. Mean sea-ice concentration (SIC) change (relative to non-cyclone reference) during cyclone clusters (panels **a**, **d**), during solitary cyclones (panels b, **e**), and the difference between clusters and solitary cyclones (cluster minus solitary, panels **c**, **f**). Top panels (**a**–**c**) indicate the cold season (NDJFM) and bottom panels (**d**–**f**) the warm season (MJJA) for 2004–2024. The light blue line indicates the position of the sea-ice edge (15% mean SIC contour). Hatching indicates grid-cells, where the signal is not statistically significant. Ice-covered grid cells are color shaded only if they have at least 5 cyclone (cluster) samples.

For the cold season, we find that cyclone clusters result in significantly greater SIC-loss than solitary cyclones throughout the Atlantic and Pacific marginal ice zone (MIZ) of the Arctic Ocean (Fig. 2a–c). Locally, the average SIC-decrease during cyclone clusters amounts to more than −15% compared to non-cyclone conditions (Fig. 2a). The difference between cyclone cluster and solitary cyclone impacts (Fig. 2c) is particularly pronounced in the Labrador Sea, the Bering Sea, the Sea of Okhotsk, and in the Barents and Kara Seas close to Svalbard and Novaya Zemlya. Averaged over the entire Arctic Ocean (north of 70^∘^N), the passage of cyclone clusters lasts 2.6 times longer than for solitary cyclones, and the resulting overall SIC reduction is 2.1 times stronger (Table 1). Similar values are found for the Eurasian Arctic and the Canadian Archipelago sector, while in the Amerasian Arctic, the amplification of cyclone cluster impacts amounts to 6.8. This remarkably strong signal is driven by particularly intense SIC-reductions due to cyclone clusters in the Bering Sea (Fig. 2). A potential explanation for this strong signal in the Bering Sea might be a wave-induced destruction of the sea-ice cover, since severe storms are known to trigger strong wave build-up in this region^48^. Further, the Eurasian and Amerasian sectors of the Arctic (the latter including the Bering Sea) are characterized by different background states of ocean heat transport and thus different sea-ice growth/melt environments, related to the Atlantic Multidecadal Oscillation (AMO), on the one hand, and the Pacific Decadal Oscillation (PDO), on the other hand. A potential contribution of such large-scale oscillations to the inter-basin differences should thus be kept in mind when interpreting the results.

**Table 1:** Domain-averaged sea-ice impact and duration

		Magnitude		Duration	
		Solitary	Cluster	Solitary	Cluster
**Cold season**	**Eurasian**	−1.8%	−3.6% (2.0)	2.4 d	6.3 d (2.6)
	**Amerasian**	−0.4%	−2.7% (6.8)	2.0 d	5.2 d (2.6)
	**Canadian**	−0.8%	−1.8% (2.3)	1.9 d	5.2 d (2.7)
	**Arctic**	−1.0%	−2.1% (2.1)	2.1 d	5.5 d (2.6)
**Warm season**	**Eurasian**	−2.4%	−3.7% (1.5)	2.1 d	5.4 d (2.6)
	**Amerasian**	−1.9%	−3.2% (1.7)	2.0 d	5.2 d (2.6)
	**Canadian**	−1.9%	−2.3% (1.2)	1.8 d	4.2 d (2.3)
	**Arctic**	−2.0%	−3.1% (1.6)	2.0 d	5.1 d (2.6)

Mean magnitude of SIC-impact during solitary cyclones and cyclone clusters, as well as mean duration of their passage (in days) for different seasons and regions. “Arctic" refers to the entire domain north of 70 degrees north. Numbers in parentheses indicate amplification factor between clusters and solitary cyclones.

Focusing on the warm season, the negative impact on SIC during cyclone clusters is markedly stronger than in the cold season in the central Arctic Ocean, East-Siberian Sea, Laptev Sea, and particularly the Beaufort Sea (Fig. 2d). On the other hand, the impact is weaker in the Barents-Kara Seas. Again, the impact of cyclone clusters is stronger than the impact of solitary cyclones (Fig. 2f). Depending on the region, the magnitude of the cyclone cluster impact is 1.2–1.7 times stronger, and their passage lasts 2.3–2.6 times longer than for solitary cyclones (Table 1). With approximately two-times (1.5-times) larger SIC-impact magnitudes in the cold (warm) season compared to 2.6-times longer event durations, our results suggest that the amplification of cumulative SIC reductions for cyclone clusters is primarily driven by the prolonged cyclonic wind forcing, rather than by consistently stronger daily SIC changes. The only exception is the Amerasian Arctic during the cold season, where a 6.8-fold increase in SIC impact magnitude combined with a 2.6-fold increase in event duration demonstrates that there is a stronger cumulative SIC-reduction per unit time for cyclone clusters compared to solitary cyclones.

### Recent changes between 1979–1999 and 2004–2024

Next, we compare the monthly impact of cyclone clusters and solitary cyclones on SIC for the period 2004–2024 to the earlier period 1979–1999. We focus on the Eurasian Arctic in the cold season—when cyclone clusters are most intense and most frequent in this sub-region (Fig. 1)—and the Amerasian Arctic in the warm season, where a noteworthy intensification of cyclone impacts on sea ice in recent years has been identified^41^. The remaining sub-chapters of the results section will therefore only discuss these two combinations of regions and seasons.

**Fig. 3:**
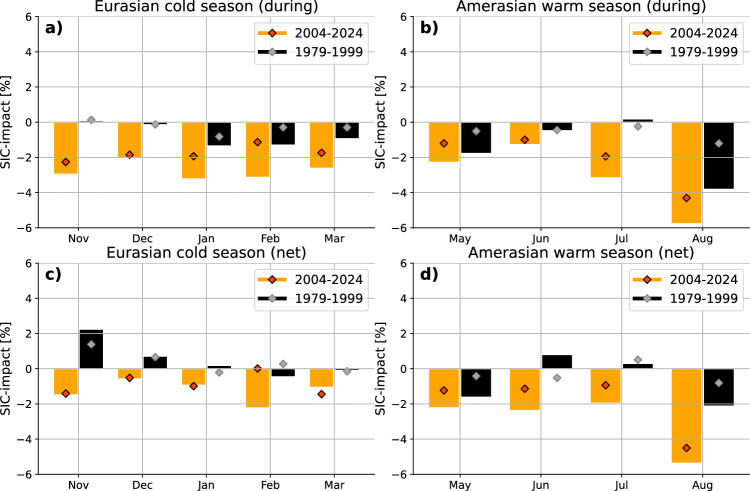
Monthly impact of cyclone clusters and solitary cyclones on sea ice for different time periods. Monthly mean sea-ice concentration (SIC) impact (relative to non-cyclone reference) for 1979–1999 and 2004–2024 for the Eurasian Arctic in the cold season (panels **a**, **c**) as well as for the Amerasian Arctic in the warm season (panels **b**, **d**). Cyclone clusters are indicated by bars, solitary cyclones by markers. Top panels (**a**, **b**) indicate SIC-impact during cyclone passages, while bottom panels (**c**, **d**) indicate “net" SIC-impact from one day before each cyclone arrival to five days after the cyclone passage.

For the Eurasian Arctic, we find consistently negative SIC-changes during cyclone clusters throughout the cold season, which have strongly amplified over the last decades (Fig. 3a). For 2004–2024, the cyclone cluster impact ranges from approx. −2% in December to −3% in January. The strongest difference compared to solitary cyclones occurs in January and February. For 1979–1999, the SIC-impact during cyclone clusters and solitary cyclones is close to zero in the early cold season (November to December), pointing to a redistribution of sea ice, where anomalous SIC decreases and increases within the cyclone area on average cancel each other out. For 2004–2024, this previously neutral signal changed to a negative one. For the late cold season (January to March), a negative SIC-impact during cyclone clusters occurs for 1979–1999 and 2004–2024, but is more than twice as strong for the more recent period.

For the Amerasian Arctic, the impact on SIC exhibits pronounced monthly differences throughout the warm season (Fig. 3b). In 2004–2024, comparatively strong SIC decreases for both solitary cyclones and clusters occur in August and July. In August, the SIC decrease during cyclone clusters amounts to −6%, which is twice as much as in the Eurasian cold season. Similar to the Eurasian cold season, cyclone cluster impacts are more intense in 2004–2024 than in 1979–1999. This is most pronounced in July. Consequently, the signal of comparatively strong SIC-loss from cyclone clusters is evident over a longer period of the melt season in 2004–2024 compared to 1979–1999, when it is basically limited to August.

Previous studies indicate that anomalous SIC-changes can persist for up to a week after the cyclone passage and in the cold season often (partially) restore the initial changes during the cyclones^29,30^. To account for this, we introduce a “net" impact on SIC, covering SIC-changes from one day before the cyclone (cluster) arrival to five days following the end of the cyclone (cluster) passage.

During 2004–2024, throughout all months of the cold season and for both solitary cyclones and clusters, the anomalous SIC-decrease during cyclones is followed by an anomalous SIC-increase after the cyclone passage (Supplementary Fig. 3). This can be explained by comparatively fast refreezing of cracks and openings in the ice cover in the cold season^29,30^. Regardless of this, for cyclone clusters, the net impact (Fig. 3c) remains negative for all months. Notably, the domain-averaged net impact of solitary cyclones is approximately in line with that of cyclone clusters, except for a major deviation for February 2004–2024. This indicates that while cold-season cyclone clusters cause a stronger and longer-lasting SIC decrease during their passage than solitary cyclones, they are also followed by a faster recovery of the ice cover. In 1979–1999, cyclone clusters caused a net increase in SIC in November through January, and decreased SIC only in February. In contrast, a net SIC decrease is found from November through March for 2004–2024. The temporal change signal is particularly strong in November, where the net cluster impact changed from approximately +2% to approx. −1.5%.

In contrast to the Eurasian cold season, most of the SIC-loss during Amerasian warm season cyclone clusters translates into a persistent ice loss, resulting in a stronger negative net impact on SIC (Fig. 3d). Compared to 1979–1999, the net cluster impact switched from (slightly) positive to negative in June and July 2004–2024, and the negative net impact on SIC in August intensified drastically. This demonstrates that the increased tendency of cyclone clusters (and solitary cyclones) to cause an overall reduction in SIC in recent years also extends to the Amerasian Arctic and the warm season.

### Relevance of cluster size and cyclone intensity

Next, we analyze how the number and intensity of clustered cyclones influence the absolute magnitude of their impact on SIC, focusing on 2004–2024 and the Eurasian cold season as well as the Amerasian warm season. We divide the analysis into MIZ (15% <= SIC <= 80%) and pack ice (SIC > 80%) conditions, as previous work revealed a strong sensitivity of a cyclone’s sea-ice impact to the initial SIC state^30,40^.

**Fig. 4:**
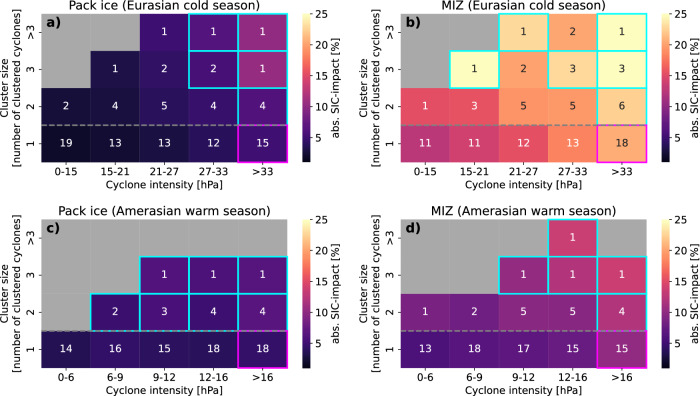
Role of cyclone intensity and the number of clustered cyclones for the absolute impact on sea ice during 2004–2024. Colors indicate the absolute value of sea-ice impact during Eurasian cold-season cyclones (**a**, **b**) and Amerasian warm-season cyclones (**c**, **d**), depending on the average cyclone intensity within a cluster and the number of clustered cyclones (One clustered cyclone indicates a solitary cyclone). Numbers in the boxes indicate the share of each category on all samples in percent; categories below 1% sample size are grayed out. Left panels (**a**, **c**) represent cases where the SIC one day before the cyclone cluster arrival is above 80% ("pack ice"), right panels (**b**, **d**) represent initial SIC between 15% and 80% ("MIZ"). Cyan bordered squares highlight categories where two or more clustered cyclones have a stronger impact than the most intense single cyclone category (pink bordered square). Cyclone intensity bin edges are defined by the 20th, 40th, 60th and 80th percentiles of the total cyclone intensity distribution.

Throughout both SIC conditions and for all seasons/regions, the absolute magnitude of the SIC-impact increases with both cyclone intensity and number of clustered cyclones (Fig. 4). In general, the impact of cyclone clusters on SIC is smaller in the pack ice than in the MIZ. For the Eurasian cold season MIZ, particularly strong impacts on sea ice are typically found when the cluster size reaches three or more cyclones (Fig. 4b). In the Amerasian warm season (Fig. 4c, d), clusters of three or more cyclones are a comparatively rare phenomenon, which fits to our previous findings (Fig. 1). While the absolute impact on SIC still increases with the number of clustered cyclones, the relation to cyclone intensity is less clear than for the Eurasian cold season. This might be because, compared to the cold-season conditions, the cyclone intensities during the warm season are relatively weak (note the difference in the intensity categories in Fig. 4).

There are two more interesting contrasts to the Eurasian cold season: For pack ice conditions, already two clustered cyclones of low-medium intensity on average exceed the effect of a single cyclone of the highest intensity class (cyan boxes in Fig. 4c). Furthermore, a contrasting seasonal and regional difference is found depending on the background sea-ice conditions. In the MIZ, the impact on SIC is smaller for the Amerasian warm season than for the Eurasian cold season (Fig. 4b, d), which can be expected due to the lower intensity of cyclones for the Amerasian warm season. In contrast, no pronounced difference between the two seasons/regions is found in pack ice (Fig. 4a, c), despite the difference in cyclone intensity. This motivates a deeper analysis of how the background sea-ice conditions modulate the impact of cyclone clusters on sea ice.

### Role of initial SIC prior to cyclone passage

**Fig. 5:**
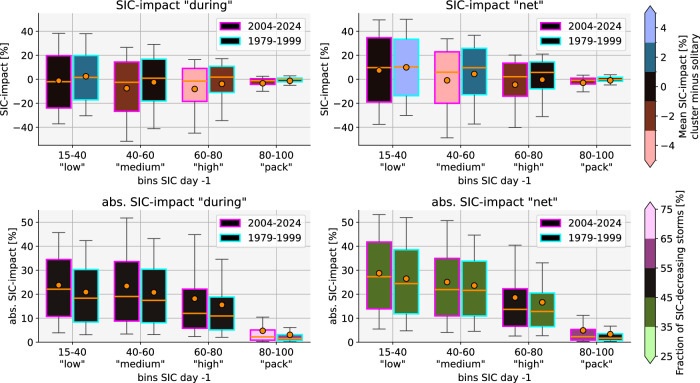
Influence of the initial ice concentration on cyclone-induced sea-ice changes in Eurasian cold season. Box-Whisker plots of the sea-ice impact during (left) and the net sea-ice impact (right) of cyclone clusters in 2004–2024 (pink-framed boxes) and 1979–1999 (cyan-framed boxes), depending on the SIC before the cyclone arrival for the Eurasian Arctic (domain outlined in Fig. 1a) and the cold season (NJDMF). Bottom panels show absolute values of the sea-ice impact. Orange circles (lines) indicate the mean (median), edges of the boxes (whiskers) indicate 25th and 75th (10th and 90th) percentiles. Box colors indicate the difference in mean SIC-impact for clusters minus solitary cyclones (top) and fraction of SIC-decreasing cyclone clusters (bottom).

**Fig. 6:**
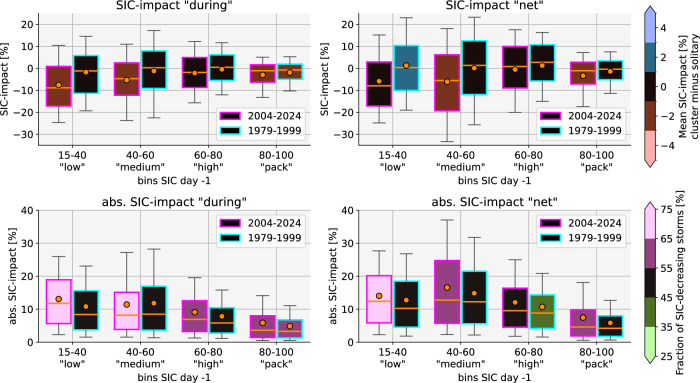
Influence of initial ice concentration on cyclone-induced sea-ice changes in the Amerasian warm season. As Fig. 5, but for the warm season (MJJA) and the Amerasian Arctic (domain outlined in Fig. 1d).

The impact of cyclone clusters on SIC is expected to depend on the characteristics of the sea ice they overpass. This dependence is illustrated in Figs. 5, 6, which depict the distribution of SIC changes during cyclone clusters for different initial SIC conditions prior to the cyclone passage. Results are presented separately for 2004–2024 (pink-framed boxes) and 1979–1999 (cyan-framed boxes).

Focusing first on the more recent period and the Eurasian cold season (pink-framed boxes in Fig. 5a), we find that the strongest SIC-reductions during cyclone clusters occur for medium and high initial SIC conditions. For high initial SIC, the strongest difference to solitary cyclones is found (indicated by box-colors in Fig. 5a). For low initial SIC, the mean impact of clusters (and solitary cyclones) is close to zero, but the large box width points to a redistribution of SIC, with positive and negative changes canceling each other out, rather than an absence of any impact. This also becomes evident when the absolute values of SIC-changes are analyzed (Fig. 5c), which are highest for low and medium SIC and gradually decrease for higher SIC categories. These findings align well with sea-ice dynamics theory, according to which the ice strength—and thereby also its capability to resist atmospheric forcing - decreases with decreasing ice concentration and thickness^49^.

The net impact of cyclone clusters (defined as SIC-change from one day before cluster arrival to five days after cluster end) is positive for low initial SIC, neutral for a medium one, and negative for high initial SIC (Fig. 5b). In alignment with our previous findings (Fig. 3), the net impact is more positive than the impact during the clusters for all SIC-conditions. The net SIC-gain for low initial SIC could be explained by advection of sea ice from regions of higher SIC. For medium to high initial SIC, the less negative net impact compared to the changes during the clusters could likewise be related to advection from surrounding areas, or to ice growth in open-water areas formed during the cluster passage.

A surprising result is that the relation between the initial SIC and the cyclone cluster impact is not constant in time, but has changed between 1979–1999 and 2004–2024 (pink-framed vs. cyan-framed boxes in Fig. 5a–d). In general, the impact in a given SIC category has increased in magnitude (Fig. 5c, d) and became more negative (Fig. 5a, b) for the more recent period. This is also reflected in the higher share of cyclones that decrease SIC (box colors in Fig. 5c, d) in pack ice conditions for 2004–2024 than for 1979–1999, considering the time during the clusters as well as the net impact. For the net impact, the higher share of SIC-decreasing cyclones is also found for high initial SIC.

For the Amerasian warm season, a strong negative impact on SIC is found for low and medium initial SIC, which is a notable contrast to the Eurasian Arctic (Fig. 6). This is true for the immediate changes during the clusters and for the net impact. Also, the strongest difference between cyclone clusters and solitary cyclones emerges from these low and medium initial SIC conditions. Again, the relation between the initial SIC and the impact of cyclone clusters has changed over time: Specifically, the intensified negative impact on low and medium initial SIC in 2004–2024, which cannot be found for 1979–1999, is a striking result. This drastic signal is accompanied by a remarkable increase in the share of SIC-decreasing cyclones from around 50% in 1979–1999 to more than 65% for low and medium SIC (box colors in Fig. 6c, d). A potential explanation could be a changed upper ocean stratification, facilitating the upward mixing of oceanic heat during cyclone passages^41^. This hypothesis fits the fact that the strongest change signal emerges in low to medium SIC conditions (Fig. 6), which are most favorable for wind-induced mixing of the upper ocean.

It has been hypothesized that intensification of cyclone-induced SIC changes in the Arctic in recent years is primarily driven by the decline in mean SIC and the associated reduction in sea-ice strength^22^. In contrast, our results indicate that cyclone (cluster) impacts on SIC have intensified in the New Arctic period even within fixed SIC conditions (Figs. 5, 6). This finding suggests that also factors other than the ongoing SIC-decline contribute to the observed intensification. To clarify this, we first examine how the composition of the Arctic sea-ice cover with respect to the previously defined SIC categories has changed between the Old and New Arctic periods. We then explicitly quantify the extent to which these shifts in SIC conditions contribute to the strengthening of cyclone-cluster impacts.

**Fig. 7:**
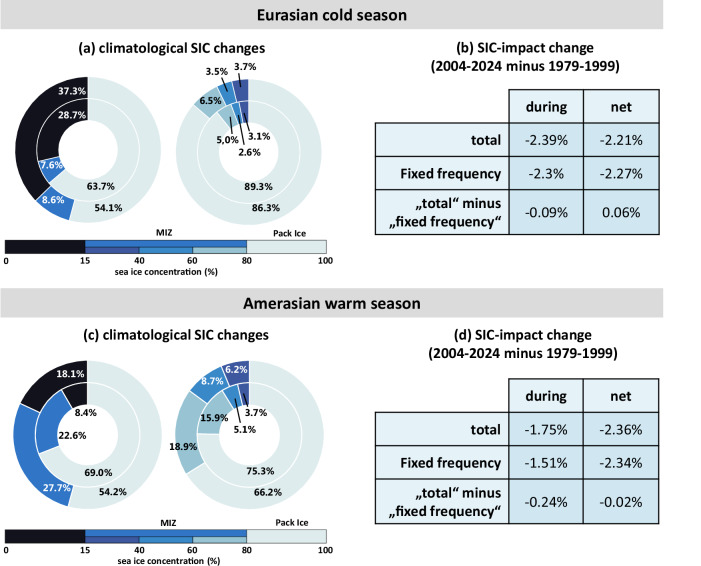
Changing sea-ice concentration composition and its relevance for recent changes in cyclone cluster impacts. Panels **a**, **c**: Mean sea-ice concentration (SIC) composition of the whole (left) and ice-covered (SIC>15%; right) domain for 2004–2024 (outer circles) and 1979–1999 (inner circles) for the Eurasian cold season (**a**) and Amerasian warm season (**c**). All changes between the two time periods are statistically significant. Panels **b**, **d**: Decomposition of the changing SIC-impact between 1979–1999 and 2004–2024 (details in “Methods” section).

For the Eurasian cold season, pack ice extent has diminished from 63.7% of the total domain area in 1979–1999 to 54.1% in 2004–2024, while the MIZ has grown by one percent (Fig. 7a). Focusing on the composition of the remaining ice cover with respect to the previously defined SIC categories, all MIZ categories have become more frequent at the expense of the pack ice, which would promote stronger cyclone cluster impacts on SIC. However, the signal is not very strong (even though it is statistically significant), with pack ice still forming the clear majority of all samples where there is SIC > 15% in 2004–2024 (86.3%).

In contrast to the Eurasian cold season, the Amerasian Arctic was nearly completely covered by sea ice in the warm season in 1979–1999 (Fig. 7c). In the more recent period 2004–2024, sea-ice decline leaves 18.1 % of the sector ice-free. In alignment with previous work, we further find a widening of the warm-season MIZ^50^ at the expense of pack ice. As a consequence, the SIC-composition of the remaining ice cover in 2004–2024 differs notably from the ice cover in 1979–1999. The share of pack ice decreased from 75.3 to 66.2%, and all MIZ categories became more frequent, specifically low and medium SIC conditions. This signal is markedly stronger than for the Eurasian cold season.

To disentangle the individual effects of the ongoing SIC-decline (Fig. 7a, c) and the intensification of cyclone impacts in a certain SIC category (Figs. 5, 6), we decompose the temporal change in the cyclone cluster impact on SIC into a part where the occurrence frequency of the individual SIC-categories is fixed to the state of 1979–1999 (“fixed frequency”)—and every change solely comes from changes within the mean impact per SIC-category—and a residual (“Total minus fixed frequency”). Details on this procedure can be found in the “Methods” section.

This analysis reveals that in the Eurasian cold season, the higher occurrence frequency of lower SIC-categories compared to pack ice contributes only weakly to the recently intensified impact of cyclone clusters (“Total minus fixed frequency”, −0.09% of the total −2.39%, Fig. 7b). Instead, most of the signal is due to intensified SIC-changes within a SIC-category (“fixed frequency”, −2.3% of the total −2.39%). For the net-impact, the contribution from changes in the occurrence frequency is similarly weak (+0.06%) and contrary to the negative overall signal, since in lower SIC-categories, more positive net impacts occur (Fig. 5). For the Amerasian warm season, approximately. 14% of the overall change in the impact during cyclone clusters can be attributed to the ongoing sea-ice decline ("Total minus fixed frequency", −0.24% of the total −1.75%, Fig. 7d). While this is a substantially larger contribution than for the Eurasian cold season, most of the intensified impact on SIC is still due to stronger impacts within fixed SIC-categories. For the net impact, again, a low relevance of the SIC-decline is found.

## Discussion

In this study, we have analyzed the impact of serial cyclone clusters on Arctic SIC, compared it to the impact of non-clustered solitary cyclones, and evaluated the relevance of cyclone cluster properties as well as the background sea-ice conditions. A key result is that clusters of two or more cyclones impact the ice approximately 2.5 times longer and cause two times stronger overall SIC-reductions than solitary cyclones (Table 1). This stronger and longer-lasting decrease in regional sea-ice area highlights the relevance of serial cyclone clustering in the Arctic: I) for the Arctic climate system, because fluxes of moisture, energy and particles (including aerosols) between the Arctic ocean and atmosphere are largely controlled by the presence of open water areas. II) for the Arctic ecosystem, because reductions in ice area enhance light availability and strengthen wind-driven mixing, with implications for nutrient availability and phytoplankton blooms^51–53^ III) for Arctic navigation, since cyclone clusters prolong disruptions where storms cause strong ice drift and deformation, generate large ocean waves, increase the risk of ice accumulation on ship superstructures, and cause navigation windows to open or close rapidly ^16,54^.

We further identified an intensification of the SIC-decreasing impact of cyclone clusters between the periods 1979–1999 and 2004–2024. Unlike expected, this intensification cannot primarily be explained by the ongoing SIC-decline in the Arctic, because in a somewhat surprising result, we find that the relationship between the initial SIC (prior to cluster arrival) and the strength of the cyclone cluster impact on SIC changed over time. Specifically, the absolute magnitude of the SIC-impact and the share of SIC-decreasing cyclones (both within fixed SIC-categories) are higher in 2004–2024 than in 1979–1999 (Figs. 5, 6). Following sea-ice dynamics theory, this could be related to the recent strong decline in Arctic SIT detected utilizing PIOMAS^55^ reanalysis data (Supplementary Fig. 4). This SIT decline should lower the ice strength and thus facilitate atmospheric impacts on sea ice^49^. Utilizing additional SIT data from the GLORYS12^56^ ocean reanalysis (see “Methods” section) for the new Arctic period (2004-2024), we indeed find that the strength of cyclone cluster impacts on SIC increases as SIT decreases below 1 m (Supplementary Fig. 5). This is particularly pronounced for the Eurasian cold season. For the Amerasian warm season, where the strongest intensification between Old and New Arctic periods has been found for low to medium SIC conditions, potential changes in upper ocean stratification facilitating the upward mixing of oceanic heat could be a further contributor to the differences between the time periods. Cyclone clusters, which prolong phases of wind-induced mixing compared to solitary cyclones, might be particularly effective in that regard. Follow-up research utilizing fully coupled models that include a dynamic ocean might be beneficial to reveal more details about potential oceanic drivers of increased SIC loss due to cyclone clusters in recent years.

Another major difference between the Old and New Arctic periods is the transition of the Atlantic Multidecadal Oscillation (AMO) from a negative to a positive phase^57^. As variations in large-scale oceanic variability such as the AMO or the Pacific decadal oscillation (PDO) control ocean heat transport into the Arctic and thus the sea-ice growth/melt environments in which the impacts of cyclone clusters take place, an analysis centered on this large-scale oceanic variability could reveal additional insights. It should further be noted that our differentiation between cyclone clusters and solitary cyclones is solely based on sea-level pressure fields. Consequently, vertical coupling processes between surface cyclones and upper-level atmospheric features - which, for example, played a key role for the Great Arctic Cyclone in 2012^58^ - are not captured in our analysis.

Focusing on the future development of the recent trends in cyclone cluster impacts on sea ice, potential changes in atmospheric circulations in the Arctic under global warming are another source of uncertainty. There is recent observational evidence of sea-ice loss during several subsequent cyclones, which have been steered into the Arctic by pronounced atmospheric blocking^59^. It has been hypothesized that such atmospheric blocking situations might become more frequent in the future due to a weaker and wavier jet stream^60^. Furthermore, there is evidence for a positive cyclone-sea ice feedback loop involving local baroclinity: In the circumpolar scale, the climatological net effect of individual cyclones and, in particular, cyclone clusters is to decrease SIC (Figs. 2, 3). During the cold season, this reduced SIC enhances turbulent heat fluxes from the ocean to the atmosphere^61^. The resulting reduced static stability increases the Eady growth rate^62^, which in turn favors enhanced cyclone activity^63^.

To conclude, we have demonstrated that serial cyclone clustering occurs with a considerable frequency in the Arctic and is related to significantly amplified sea-ice loss compared to solitary cyclones. Thus, cyclone clustering can be considered a highly relevant mechanism for Arctic cyclone-sea ice interactions. More generally, our results highlight the added value of taking into account the timing of weather events relative to each other and possible constructive interferences among what are typically considered independent events. Applying similar frameworks to other target quantities (e.g., snow accumulation on sea ice or wind-driven ocean currents) and further types of weather events could thus help to further sharpen our understanding of the role of weather extremes in the coupled polar climate system.

## Methods

### Data

This study utilizes 6-hourly sea level pressure (SLP) and daily sea-ice concentration (SIC) data from the ERA5 reanalysis^64^. SIC fields in ERA5 are derived from satellite data (HadISST2 and OSI SAF). Both SLP and SIC fields are provided on a regular longitude-latitude grid with 0.28125 degrees horizontal resolution. To avoid errors during the application of the cyclone tracking algorithm (see below) in the polar regions, SLP data is re-gridded to a polar azimuthal equal-area projection (25 km resolution EASE-Grid 2.0^65^) using bilinear interpolation. To maintain spatial consistency between both variables, SIC data is treated accordingly.

Further, sea-ice thickness (SIT) data from the Pan-Arctic Ice Ocean Modeling and Assimilation System PIOMAS^55^ as well as SIT and SIC data from the CMEMS global ocean eddy-resolving reanalysis GLORYS12^56^ version 1 (hereafter GLORYS12) are utilized. PIOMAS data are used to assess long-term changes in SIT between the Old Arctic and New Arctic periods (1979–1999 and 2004–2024, respectively). GLORYS12, which is only available from 1993 onward but is more advanced in terms of model physics, resolution, and data assimilation, is used to analyze the role of sea-ice thickness in modulating cyclone-induced SIC changes during the New Arctic period. The qualitative similarity in the SIC response to cyclones in ERA5/satellite data and in GLORYS12 demonstrates that GLORYS12 is suitable for this purpose (Supplementary Fig. 6).

### Target seasons and regions

In this study, we contrast anomalous SIC-changes associated with cyclones in the cold season (November to March) and warm season (May to August). The specific grouping of months is designed to differentiate between seasons when the SIC in the Arctic is usually increasing or decreasing due to sea-ice growth or melt (Supplementary Fig. 7). This facilitates the interpretation of relative SIC-change signals for cyclones compared to non-cyclone conditions. October is not included in the cold season because, relative to the subsequent months, the mean sea-ice extent is still lower and the climatological increase in SIC (in the absence of cyclones) is more strongly pronounced, specifically along the Siberian coast (Supplementary Fig. 7). In addition to a pan-arctic analysis, more details are presented for two focus regions, the “Eurasian Arctic" (0–140^∘^E and 67.5–90^∘^N, domain outlined in Fig. 1a), and the “Amerasian Arctic" (140–240^∘^E and 70–90^∘^N, domain outlined in Fig. 1d). The choice of these regions is motivated by previous work that points to different mechanisms of cyclone impacts on sea ice in the two domains^41^.

### Cyclone tracking and cluster detection

The tracking of cyclones was conducted with the Multi-Object Analysis of Atmospheric Phenomena (MOAAP) program^47^. Cyclones are identified as negative SLP anomalies of at least -8 hPa strength (standard parameter setting of MOAAP). To calculate this SLP anomaly, the 6-hourly SLP field from ERA5 is first smoothed with a uniform square filter with a length of 100 km to remove small-scale noise and local orographic effects, and afterward compared to a background SLP environment obtained by applying a uniform square filter with a side length of 3000 km and a temporal extent of 78 h^47^. The output of the tracking algorithm consists of a binary cyclone occurrence field ("cyclone shapes") as well as a set of cyclone properties (including size, intensity, etc.) for each tracked cyclone object. The cyclone shapes are used to identify periods during which a grid-cell was situated within the area of two different, subsequent cyclones with no more than 48 h between the cyclone occurrences. If this criterion is fulfilled, this is considered a cyclone cluster event. If additional subsequent cyclones occur within 48 h, the cyclone cluster is extended until no further cyclone occurrences are detected for at least 48 h. In accordance with that, the criterion for a solitary cyclone is that no other cyclones occur for 48 h prior to and after the cyclone passage. Cyclone intensity is calculated for cyclone clusters and solitary cyclones as maximum minus minimum SLP within the cyclone area for each time step of the cyclone lifetime. When cyclone intensity is related to the cyclone impact on SIC (see below), it is averaged for the time of the cyclone (cluster) occurrence at the respective sea-ice covered grid-cell. For cyclone clusters, this means that the cluster intensity is the average intensity over all cyclones within the cluster.

### Impact of cyclones on SIC

The method for determining the impact of cyclones on SIC is based on previous work^30^. The following two steps are conducted whenever a sea-ice covered grid-cell is situated within a cyclone: 1) the change in SIC is calculated from one day before the first cyclone occurrence to the last day of the cyclone occurrence; 2) a non-cyclone reference SIC-change (see below) for the same number of days is subtracted. That way, anomalous SIC-changes during cyclones are determined. The same procedure is repeated for a time period of 5 days following each cyclone (last day of cyclone occurrence to five days later), to quantify anomalous SIC-changes after cyclones, e.g., enhanced sea-ice growth due to refreezing of cracks and openings in the ice under cold conditions on the backside/western flank of a cyclone^30^. By combining the time periods during and after each cyclone, a “net” impact on SIC is determined as anomalous SIC-change from one day before the first cyclone arrival to 5 days after the end of the cyclone. Regional mean impacts on SIC (e.g., in Table 1) are calculated as spatial averages over all (ice-covered) grid-cells of the respective domains. Thus, cyclones that pass over grid-cells in not just one but multiple domains during their lifetime contribute to the regional mean SIC impacts in multiple domains. The same applies when regional mean SIC impacts are calculated for different categories of initial sea-ice conditions.

### Non-cyclone reference SIC-change

The non-cyclone reference is based on the average four-day SIC-change at a grid-cell for all time steps, when the grid-cell was not situated within a cyclone for six consecutive days (grid-cell was cyclone-free from one day before to one day after the four-day period). For each specific cyclone occurrence, the non-cyclone reference is scaled in time to match the exact cyclone duration. For example, if a cyclone passage lasts for 2 days, the 4-day non-cyclone reference SIC-change is divided by two. The non-cyclone reference is calculated separately for each calendar month and for the periods 1979–1999 and 2004–2024. Each cyclone event is then compared against the corresponding monthly reference.

### Decomposition of the changed (2004–2024 minus 1979–1999) SIC-impact

To disentangle the contributions of intensified cyclone impacts on SIC within fixed SIC-categories (Figs. 5, 6) and changes in the occurrence frequency of certain SIC-categories (Fig. 7) to the overall intensification of cyclone impacts on SIC, we apply the following decomposition: First, we determine the sample size and the mean SIC-impact for each SIC-category within the periods 1979–1999 (denoted old Arctic) and 2004–2024 (denoted new Arctic). Then, we calculate the mean SIC-impact over all SIC-categories for both time periods as the sum of the mean SIC-impact per SIC-category multiplied by the fraction of the category sample size on the overall sample size. The difference between the two time periods (2004–2024 minus 1979–1999) is referred to as “total” (Eq. (1)). Afterward, we calculate the respective difference in overall SIC-impact for “fixed frequency” (“ff”), where for the period 2004–2024, the actual sample size of each SIC-category is replaced with the sample size of 1979–1999, while the mean SIC-impact per category still originates from 2004-2024 (Eq. (2)).

1 $$\overline{S{I}_{diff,total}}={\sum }_{c={c}_{1}}^{{c}_{n}}\frac{{N}_{c,new}}{{N}_{new}}\overline{S{I}_{c,new}}-{\sum }_{c={c}_{1}}^{{c}_{n}}\frac{{N}_{c,old}}{{N}_{old}}\overline{S{I}_{c,old}}\,,$$

2 $$\overline{S{I}_{diff,ff}}={\sum }_{c={c}_{1}}^{{c}_{n}}\frac{{N}_{c,old}}{{N}_{old}}\overline{S{I}_{c,new}}-{\sum }_{c={c}_{1}}^{{c}_{n}}\frac{{N}_{c,old}}{{N}_{old}}\overline{S{I}_{c,old}}\,,$$

where *SI* is the SIC-impact of cyclones, *N* is the sample size and *c* represents the SIC-categories “low”, “medium”, “high” and “pack”.

The SIC-impact of “fixed frequency" indicates the change in overall SIC-impact solely due to the intensified mean SIC-impact within each SIC-category. Finally, we calculate the difference between “total" and “fixed frequency", which indicates the additional change in overall SIC-impact due to the changing occurrence frequency of each SIC-category (and thus the ongoing sea-ice decline in the Arctic).

### Statistical significance

Throughout the manuscript, statistical significance is reported at the 95% level based on a Student’s *t*-test.

## Supplementary information

Supplementary Information

Transparent Peer Review file

## Data Availability

The datasets generated during and/or analysed during the current study are available in public repositories: • The ERA5 reanalysis on single levels is available at the Copernicus Climate Data Store: 10.24381/cds.adbb2d47^66^. • The cyclone tracking output (including the cyclone cluster climatology) based on ERA5 sea-level pressure and the MOAAP algorithm^47^ is available at Zenodo: 10.5281/zenodo.18400260^67^. • Sea-ice thickness data from the PIOMAS reanalysis are available through the Polar Sci. Center, University of Washington: https://pscfiles.apl.uw.edu/zhang/PIOMAS/data/v2.1/^55^. • Sea-ice concentration and thickness data from the GLORYS12 version 1 ocean reanalysis are available through the Marine Data Store (MDS) of the E.U. Copernicus Marine Service Information (CMEMS): 10.48670/moi-00021^68^.

## Code availability

The computer code for conducting the analysis and generating the presented figures was created in the Python programming language and is archived at Zenodo: 10.5281/zenodo.20556452.
