# Supplementary material for: On the relevance of serial cyclone clustering for Arctic sea ice
Source: Nat Commun. 2026 Aug 5;17:7847. doi: 10.1038/s41467-026-76245-5 (PMC13443582; doi:10.1038/s41467-026-76245-5)
Supplement: Supplementary file 1 [file 41467_2026_76245_MOESM1_ESM.pdf]

Supplementary information for manuscript "On the relevance of serial cyclone clustering for Arctic sea ice"

Lars Aue[1*], Sofie Tiedeck[1], Peter Finocchio[2], Timo Vihma[3], Petteri Uotila[4], Gunnar Spreen[5], Annette Rinke[1]

[1]Alfred Wegener Institute, Helmholtz Center for Polar and Marine Research, Potsdam, Germany..
[2]Naval Research Laboratory (NRL), Monterey, California, USA..
[3]Finnish Meteorological Institute, Helsinki, Finland..
[4]Institute for Atmospheric and Earth System Research/Physics, Faculty of Science, University of Helsinki, Helsinki, Finland..
[5]University of Bremen, Institute of Environmental Physics, Bremen, Germany..

*Corresponding author(s). E-mail(s): lars.aue@awi.de;

1 Supplementary Figures

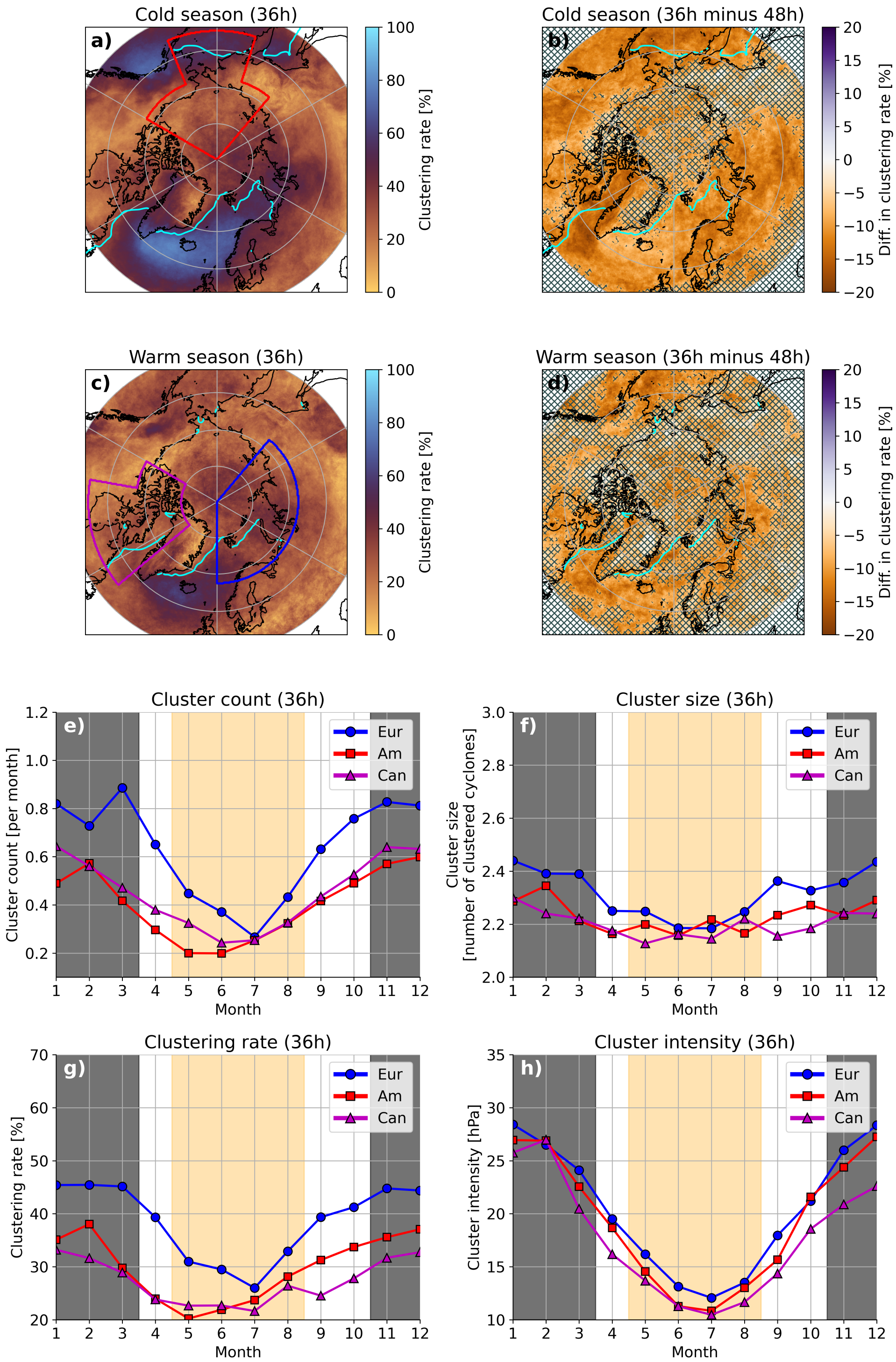

Supplementary Fig. 1 Occurrence and properties of serial cyclone clusters in the Arctic for varied clustering threshold. Panels a and c indicate clustering rate (share of all cyclones which occur within clusters) for the cold season (NDJFM, a) and warm season (MJJA, c) for the years 2004-2024 with a threshold of 36-hours to detect serial cyclone clusters, and panels b and d indicate the respective difference to the clustering rate obtained for a 48-hour threshold. The light blue lines indicate the position of sea-ice edge (15% mean SIC contour), hatching indicates non significant signals. The remaining panels indicate the annual cycle of cyclone cluster count (e), cluster size (f), clustering rate (g) and intensity (h) for the Eurasian Arctic, the Amerasian Arctic, and the Canadian Archipelago sector (domains outlined in panels a and c). Gray and yellow shadings highlight months in the cold and warm seasons, respectively.

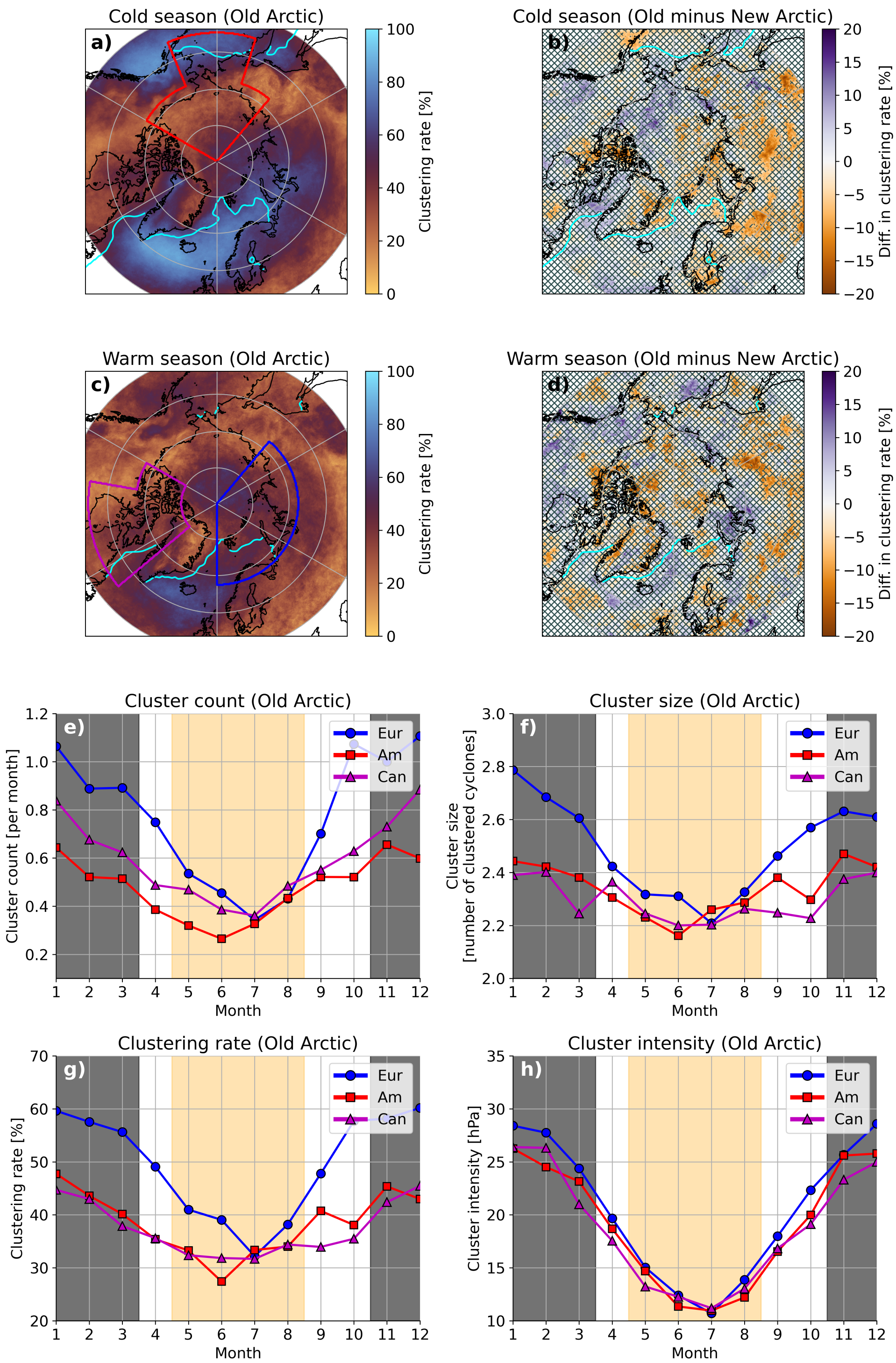

Supplementary Fig. 2 Occurrence and properties of serial cyclone clusters in the Arctic for 1979-1999. Panels a and c indicate clustering rate (share of all cyclones which occur within clusters) for the cold season (NDJFM, a) and warm season (MJJA, c) for the years 1979-1999 with a threshold of 48-hours to detect serial cyclone clusters, and panels b and d indicate the respective difference to the clustering rate obtained for 2004-2024. The light blue lines indicate the position of sea-ice edge (15% mean SIC contour), hatching indicates non significant signals. The remaining panels indicate the annual cycle of cyclone cluster count (e), cluster size (f), clustering rate (g) and intensity (h) for the Eurasian Arctic, the Amerasian Arctic, and the Canadian Archipelago sector (domains outlined in panels a and c). Gray and yellow shadings highlight months in the cold and warm seasons, respectively.

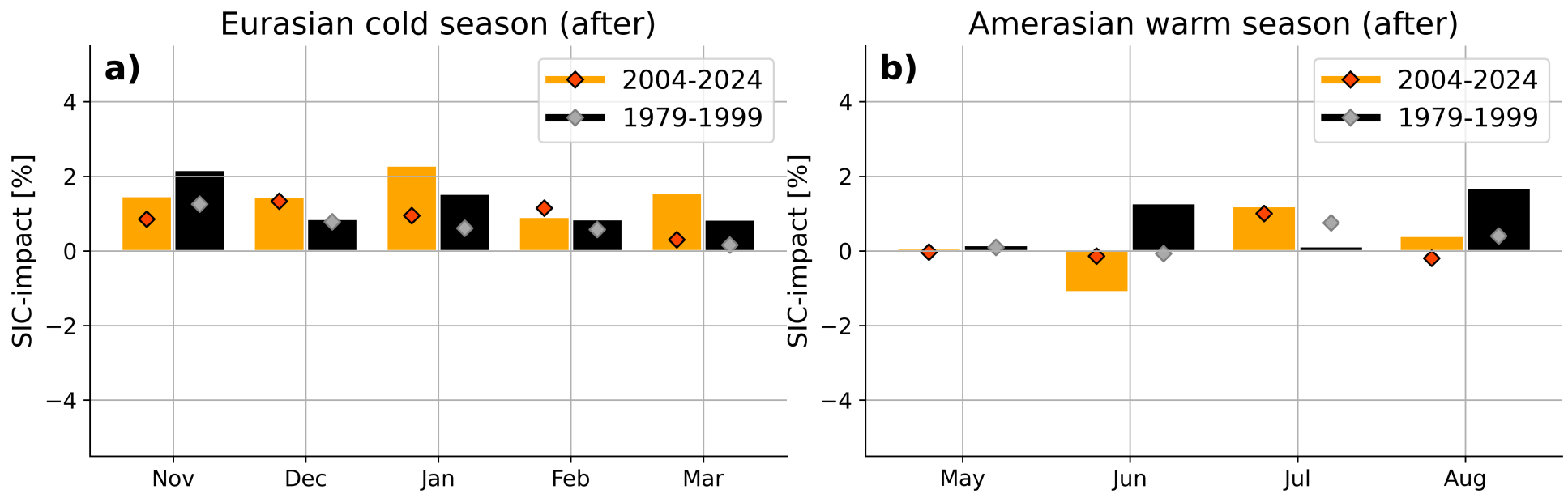

Supplementary Fig. 3 SIC-impact following cyclone clusters and solitary cyclones on SIC for different time periods. Monthly mean SIC-impact (relative to non-cyclone reference) for the five days following a cyclone event (denoted "after") for 1979-1999 and 2004-2024 for the Eurasian Arctic in the cold season (panel a) as well as for the Amerasian Arctic in the warm season (panel b). Cyclone clusters are indicated by bars, solitary cyclones by markers.

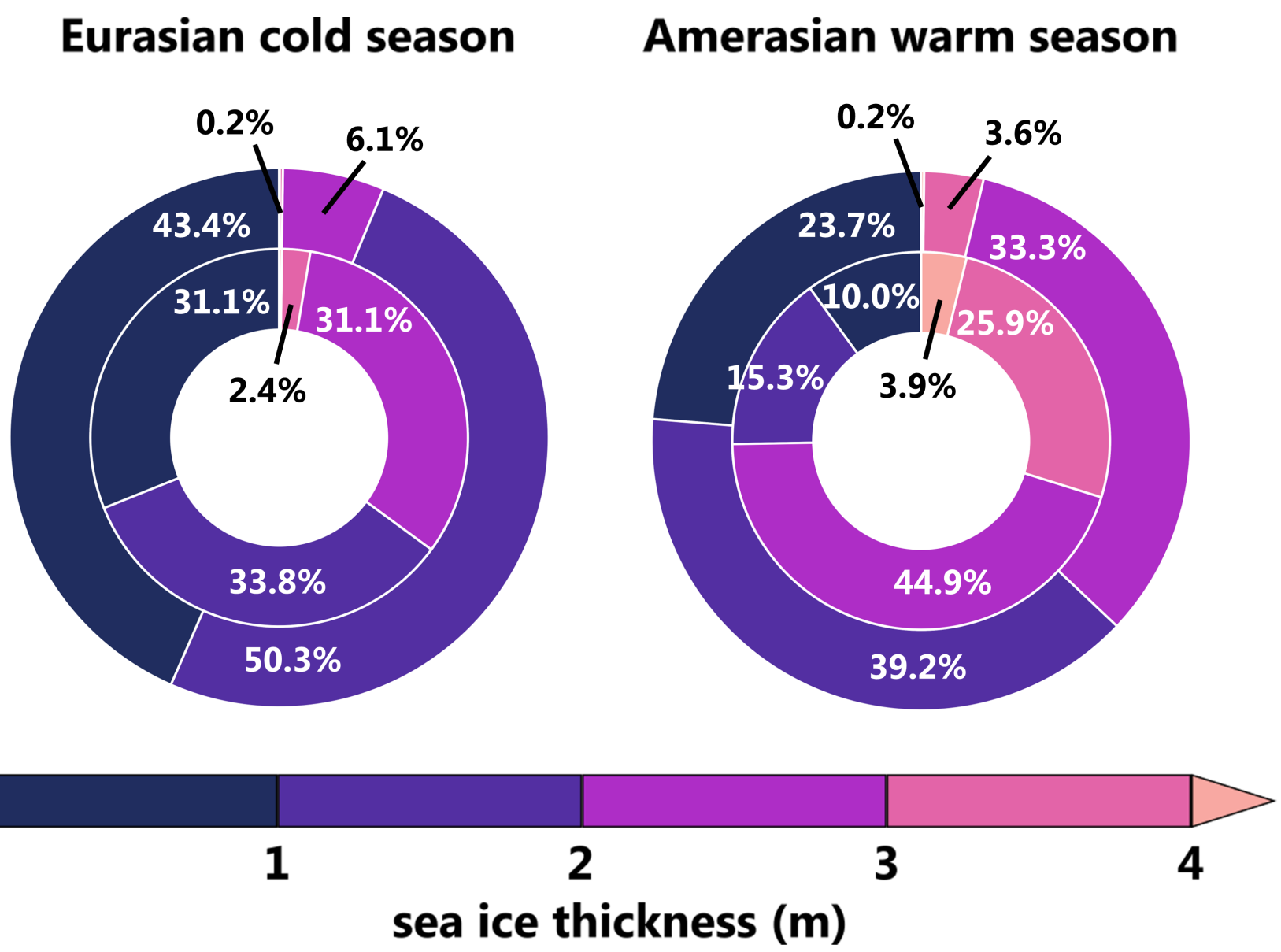

Supplementary Fig. 4 Changing sea-ice thickness composition for the Eurasian cold season and Amerasian warm season. Mean effective sea-ice thickness (which is sea ice volume by grid cell area) composition for the Eurasian cold season (left) and Amerasian warm season (right) for 2004-2024 (outer circles) and 1979-1999 (inner circles) based on the PIOMAS reanalysis [1]. All changes between the two time periods are statistically significant.

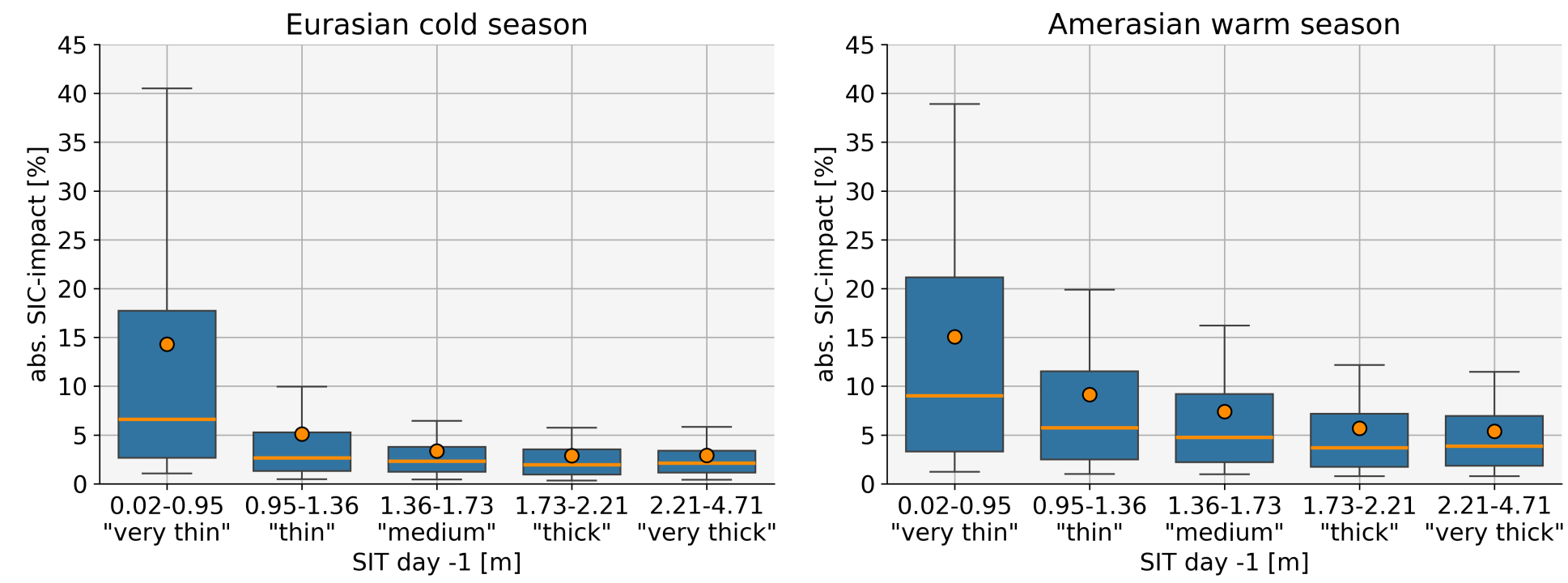

Supplementary Fig. 5 Influence of sea-ice thickness on sea-ice concentration response to cyclone clusters. Box-Whisker Plot of absolute sea-ice concentration (SIC) changes during cyclone clusters for different categories of initial sea-ice thickness (SIT) one day before the cyclone cluster arrival at an ice-covered grid-cell. Dots indicate the mean value, horizontal lines the median value, and box (whisker) edges indicate the 25th/75th (10th/90th) percentiles. SIT-bins are defined by the 0th, 20th, 40th, 60th, 80th and 100th percentile of the pan-arctic SIT distribution. The left panel shows results for the Eurasian Arctic during the cold season (NDJFM), while the right panel shows results for the Amerasian Arctic during the warm season (MJJA), both for the New Arctic period 2004-2024. SIC and SIT data originate from the GLORYS12 version 1 ocean reanalysis[2].

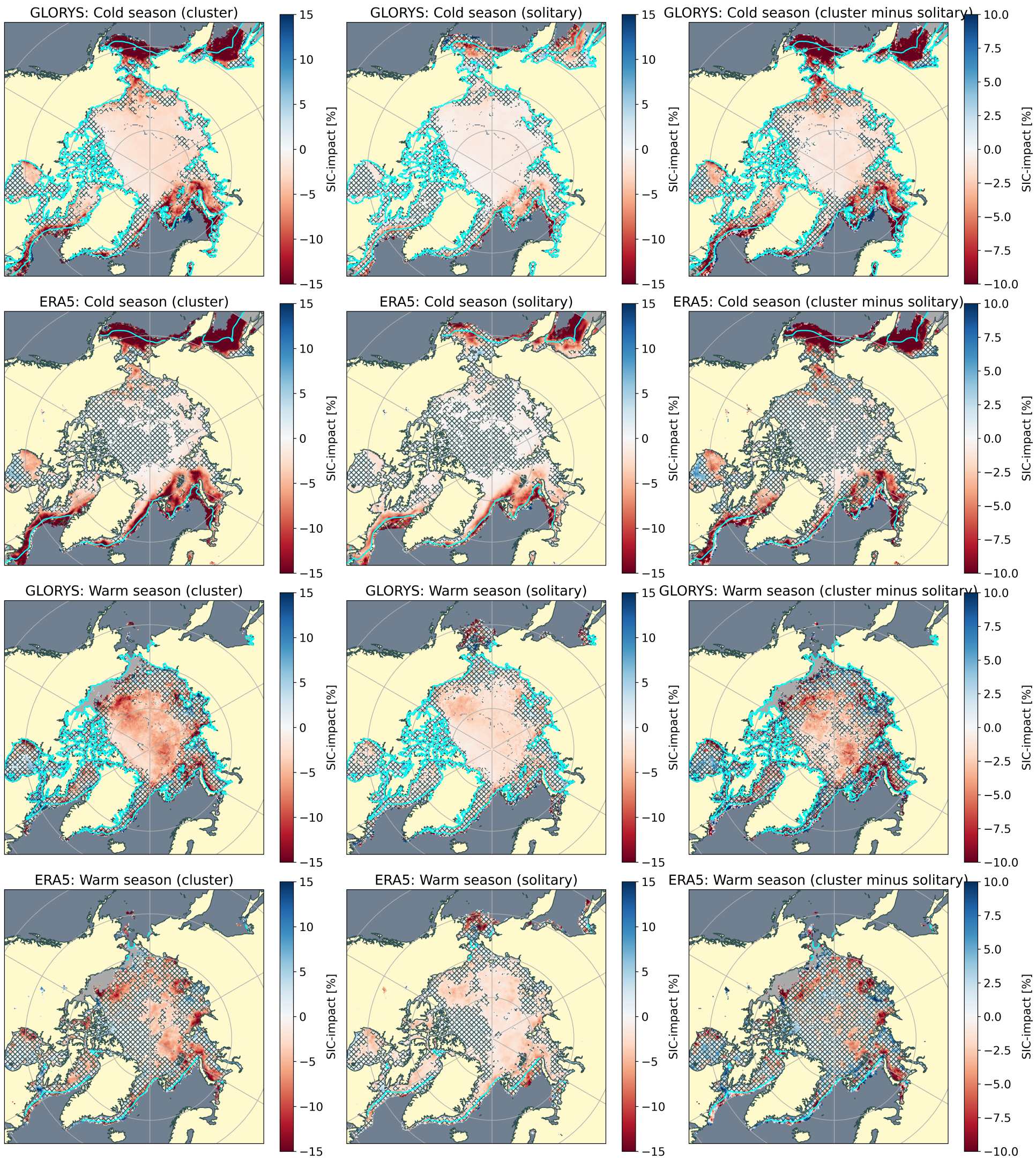

Supplementary Fig. 6 Evaluation of Impact of cyclone clusters and solitary cyclones on sea ice in GLORYS12. Mean SIC-change (relative to non-cyclone reference) during cyclone clusters (left column), during solitary cyclones (middle column), and the difference between clusters and solitary cyclones (cluster minus solitary, right column). The first row indicates the cold season (NDJFM) and GLORYS12 data[2], the second row indicates the cold season (NDJFM) and ERA5 data[3], the third row indicates the warm season (MJJA) and GLORYS12 data, and the bottom row indicates the warm season (MJJA) and ERA5 data. All panels show New Arctic period from 2004-2024. Light blue line indicates position of sea-ice edge (15% mean SIC contour). Hatching indicates grid-cells, where the signal is not statistically significant. Ice-covered grid cells are color shaded only if they have at least 5 cyclone (cluster) samples.

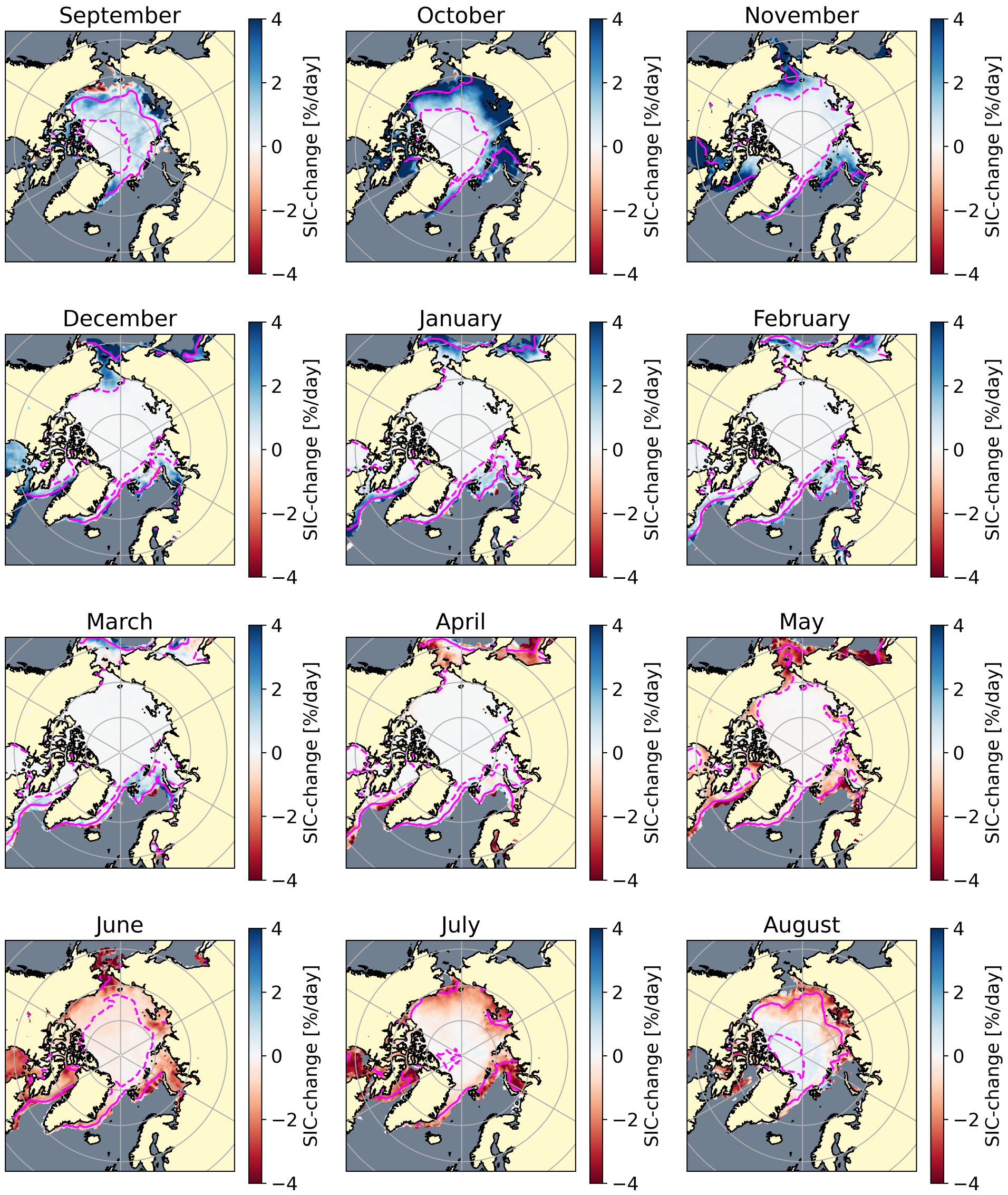

Supplementary Fig. 7 Non-cyclone reference SIC-change. Non-cyclone reference SIC-change for each month of the years 2004-2024. Solid (dashed) magenta line indicates 15% (90%) mean SIC contour.

References

[1] Zhang, J., Rothrock, D.A.: Modeling global sea ice with a thickness and enthalpy distribution model in generalized curvilinear coordinates. Monthly Weather Review **131**(5) (2003) https://doi.org/10.1175/1520-0493(2003)131⟨0845:MGSIWA⟩2.0.CO;2

[2] Jean-Michel, L., Eric, G., Romain, B.-B., Gilles, G., Angélique, M., Marie, D., Clément, B., Mathieu, H., Olivier, L.G., Charly, R., Tony, C., Charles-Emmanuel, T., Florent, G., Giovanni, R., Mounir, B., Yann, D., Pierre-Yves, L.T.: The copernicus global 1/12° oceanic and sea ice glorys12 reanalysis. Frontiers in Earth Science **Volume 9 - 2021** (2021)

[3] Hersbach, H., Bell, B., Berrisford, P., Biavati, G., Horányi, A., Muñoz Sabater, J., Nicolas, J., Peubey, C., Radu, R., Rozum, I., Schepers, D., Simmons, A., Soci, C., Dee, D., Thépaut, J.-N.: ERA5 hourly data on single levels from 1940 to present. Copernicus Climate Change Service (C3S) Climate Data Store (CDS) (2023)
