# Supplementary material for: On the relevance of serial cyclone clustering for Arctic sea ice
Source: Nat Commun. 2026 Aug 5;17:7847. doi: 10.1038/s41467-026-76245-5 (PMC13443582; doi:10.1038/s41467-026-76245-5)
Supplement: Supplementary file 2 [file 41467_2026_76245_MOESM2_ESM.pdf]

nature portfolio

Peer Review File

On the relevance of serial cyclone clustering for Arctic sea ice

Corresponding Author: Dr Lars Aue

This file contains all reviewer reports in order by version, followed by all author rebuttals in order by version.

Attachments originally included by the reviewers as part of their assessment can be found at the end of this file.

Version 0:

Reviewer comments:

Reviewer #1

(Remarks to the Author)
General Comments:

This study investigates the impact of serial cyclone clusters on Arctic sea-ice concentration (SIC), in comparison to solitary cyclones. Cyclone clusters produce a SIC reduction that is about twice as strong and lasts approximately 2.5 times longer than that of solitary cyclones. The SIC response has intensified from 1979–1999 to 2000–2024, indicating a changing relationship with background ice conditions beyond simple SIC decline.

Moreover, the impacts exhibit clear regional differences between the Eurasian and Amerasian sectors, highlighting spatial heterogeneity in cyclone–sea ice interactions. This is a novel attempt and presents an interesting analysis. However, several issues need to be addressed, including the choice of regions, comparisons across different periods, the influence of ocean heat transport and decadal variability, and the role of sea-ice thickness. In its current form, the study is not yet suitable for publication.

Major Comments:
1. Choice of regions
Although this study focuses on the Arctic Ocean, the comparison between the Eurasian and Amerasian sectors is not equivalent. The Eurasian sector (Fig. 1a) always includes the ice edge and open-ocean areas (e.g., the Barents and Norwegian Seas) and is geographically well positioned to capture the Icelandic Low, particularly in winter. In contrast, the Amerasian sector (Fig. 1d) is almost entirely covered by sea ice during winter and is located far north of the main center of cyclone activity associated with the Aleutian Low.
If regions extending to the ice edge are to be considered, the Bering Sea and the Sea of Okhotsk should be included, where strong responses are in fact noted in the manuscript (line 111, Fig. 2c). A similar argument applies to the Labrador Sea. Therefore, in order to enable a fair seasonal comparison of regions in this study, it is necessary to redefine the domains with consideration of the southern boundary of sea ice. At a minimum, an Amerasian sector encompassing the Bering and Okhotsk Seas, as well as a Canadian Archipelago sector encompassing the Labrador Sea, should be introduced.

2. Choice of periods
The manuscript compares two periods, 1979–1999 and 2000–2024. A major difference between these periods is the transition of the Atlantic Multidecadal Oscillation (AMO) from a negative to a positive phase (e.g., Dima et al., 2025, https://doi.org/ 10.1038/s41612-025-00960-x). During the positive AMO phase (2000–2024), the Icelandic Low is expected to be stronger (although it is unclear whether this directly increases the number of cyclone clusters), and, at least, ocean heat transport (OHT) toward the Arctic—favorable for suppressing sea-ice growth—should be enhanced. Therefore, consideration of the AMO appears essential, particularly for the discussion of winter conditions in the Eurasian sector.

Alternatively, if the analysis is based on climate regimes, one could classify the Eurasian sector by AMO phase, while the Amerasian sector might be better categorized by the Pacific Decadal Oscillation (PDO). The PDO has transitioned from a positive phase in the 1980s toward a more negative phase in recent decades. In contrast to the Eurasian sector, this suggests conditions that are generally less favorable for sea-ice reduction (although PDO+ can also be associated with sea-

ice loss in some cases).

In other words, a direct comparison between the Eurasian and Amerasian sectors may not be appropriate even before considering whether cyclones are solitary or clustered, because the background states of ocean heat transport—and thus the sea-ice growth environment—are fundamentally different. This raises the question of whether a simple period-based comparison is suitable, or whether an analysis centered on large-scale oceanic variability (e.g., AMO+ vs. PDO+, AMO- vs. PDO-) would yield different conclusions.

3. Treatment of cyclones across the Eurasian and Amerasian sectors
Consider the Great Arctic Cyclone mentioned in the Introduction (lines 28–30). This cyclone traverses from the Eurasian sector to the Amerasian sector as defined in this study. In such cases, clarification is needed on how these cyclones are classified—i.e., which sector they are assigned to. Furthermore, according to Yamazaki et al. (2015, 10.1002/2014JD022925), this event developed through the coupling of a surface cyclone over the Beaufort Sea with an upper-tropospheric Polar Vortex advected from the Eurasian sector. From this dynamical perspective, it is unclear whether such a case should be categorized as a solitary cyclone or as part of a cluster.

Since the cyclone tracking in this study is based on sea level pressure (SLP), it relies on detecting and following surface cyclones. However, to fully capture the dynamical structure of such events—including vertical coupling processes—it may be necessary to also consider fields at 500 hPa or 300 hPa. This limitation of the analysis should be explicitly addressed in the Discussion. In addition, it would be helpful to clarify how the Great Arctic Cyclone is categorized within the framework of this study (e.g., in Fig. 4). Providing a concrete example would also strengthen the connection to the Introduction.

4. Sea-ice thickness (SIT)
The role of SIT is briefly mentioned in the Discussion (lines 274–278), but it is not sufficiently explored. There is clear potential to incorporate SIT information from ocean reanalyses, climate model outputs, or satellite-based observations. The dynamical response of sea ice to wind forcing strongly depends on SIT and is therefore crucial for interpreting the results of this study. In addition, from the perspective of OHT, wind-driven processes that suppress sea-ice growth are also closely linked to SIT. Without adequately addressing these aspects, the mechanisms underlying the reported SIC changes remain unclear. In this sense, the manuscript provides an incomplete interpretation, as the lack of a thorough discussion of SIT limits the ability to explain the physical processes behind the presented results.

Minor comments:
1. Title: It would be more accurate to refer to *Arctic sea-ice extent* rather than *Arctic sea ice* in the title.

2. Figs. 5 & 6: They are hard to understand.

Reviewer #2

(Remarks to the Author)
Please see the attached review.

Reviewer #3

(Remarks to the Author)
Review of NCOMMS-26-009040
Title: “On the relevance of serial cyclone clustering for Arctic sea ice”
Authors: Lars Aue, Sofie Tiedeck, Peter Finocchio, Timo Vihma, Petteri Uotila, Gunnar Spreen, and Annette Rinke

Summary:

This study quantifies the effect of Arctic cyclones that occur in series, addressing an important gap in the understanding of storm effects in the polar regions. Notable differences between warm and cold season impacts and storm effects in the “new” and “old” Arctic are presented. This paper assesses important variables including the occurrence, size, and intensity of cyclone clusters, as well as changes in the underlying sea ice concentration (SIC) field between time periods. Ultimately, the authors find the impacts of cyclone clusters to be greater than solitary storms and that there is an intensification of SIC-loss due to cyclone clusters from 1979-1999 to 2000-2024, which cannot solely be explained by the changing SIC distribution in the Arctic. I found the decomposition of SIC decline and intensification of cyclone impacts to be a very interesting novel result, which, (as the authors point out in the Discussion) opens new lines of research related to the effects of sea ice thickness and upper ocean mixing on sea ice changes in the Arctic. This work provides important context for other studies on Arctic cyclone impacts and sea ice decline, highlighting the significance of considering cyclone clustering on SIC.

The paper is clearly written, and the introduction provides thorough context with appropriate references for the results that follow. The figures are very effective, and the analysis presented here is generally well thought out with the results supporting the claims made. The data and methodology are also well described, and the work could be reproduced. I believe the research is noteworthy and deserving of publication in Nature Communications. I have a few small suggestions (listed below), which are relatively minor and should hopefully not require too much new analysis or significant changes to the manuscript.

Major Comments:

My understanding of the methodology is that SIC differences are computed from one day before the first cyclone in a cluster to the last day of the final cyclone in the cluster. For the values in Table 1, it makes sense that the clusters have about 2.5 times the duration of solitary cyclones because there are more passing storms, however since the magnitude of SIC impact is only about twice as large as solitary cyclones, do clustered storms have a smaller effect per day on the ice than solitary storms? I would appreciate some clarification on the interpretation of these values.

Related to this, I wonder if it's possible to include in Table 1 the difference in SIC impacts between the first storm of the cluster and the following 1-2 cyclones to identify any "preconditioning" the first storm might do to the ice (where the later storm might have a larger or smaller impact than the previous storm). I recognize this is an additional analysis that may not fit within this paper, but just an idea to better compare cyclone impacts on similar (one storm) timescales.

Line-by-Line Comments:

17-18, 112, 119-120, 260: Along with my general comment above, the end of these sentences seems to imply that the cyclones impacts are twice as large for each day of the 2.5 times longer duration, which may not be the correct interpretation.

19: If there's room in the abstract, it might be nice to highlight that these intensified SIC losses occur even within fixed SIC categories.

75, 84: This might be somewhat superfluous because I recognize the differences are quite small, but could a third row be added to Supplementary Figs. 1 and 2 showing the differences from Fig. 1 to make the comparisons a bit easier?

118: I'm intrigued by the regions of positive SIC change in Fig. 2d in the Chukchi Sea and by the Canadian archipelago. Do you have any insight to why clusters would have a less negative ice impact than solitary cyclones in those regions?

Fig. 5: This is an interesting figure! I'm finding it a little difficult to discern the colors of the boxes (particularly near the center of the scale and for the pack ice categories) given all the additional information on the plot. Perhaps the values of the colors could be labeled along the x-axis or at the top of the whiskers.

Supplementary Figs. 4, 6: Could the respective Eurasian and Amerasian domains be added for easier comparison?

316-318: Is April excluded for a similar reason?

346: Please clarify over what time interval the non-cyclone reference period is averaged over. Are separate references used for the 1979-1999 and 2000-2024 time periods?

Version 1:

Reviewer comments:

Reviewer #1

(Remarks to the Author)
The authors have adequately addressed my comments and revised the manuscript accordingly. The manuscript is now suitable for publication.

(Remarks on code availability)

Reviewer #2

(Remarks to the Author)
Review of NCOMMS-26-00940A

Title: On the relevance of serial cyclone clustering for Arctic sea ice

Author(s): Lars Aue, Sofie Tiedeck, Peter Finocchio, Timo Vihma, Petteri Uotila, Gunnar Spreen, and Annette Rinke

Summary and Key Results: This manuscript aims to quantify the impact of cyclone clusters on very rapid sea ice loss events in the Arctic. Cyclone clusters occur when there are several cyclones that follow in near spatial and temporal proximity to each other. The analysis is conducted using ERA5 atmospheric reanalysis, ERA5 sea ice concentration (SIC), and a freely available cyclone tracking algorithm. The authors find that cyclone clusters contribute to significantly more loss in SIC over a longer amount of time compared to single (solitary) cyclone events. They also find that the losses in SIC have increased in recent times.

I thank the authors for carefully and adequately considering my comments on the previous
version. I think this article is nearly ready for publication and will make an excellent contribution to Nature Communications.

Minor edits:
1) Figure 4 caption: indicate share → indicate the share
2) 337: pronounced stronger → more strongly pronounced
3) 387, 388: Comma after equation, no indent before where on following line

(Remarks on code availability)

Reviewer #3

(Remarks to the Author)
I appreciate the authors' thorough responses and effort in improving and clarifying the manuscript. My concerns have been addressed, and I have no further comments.

(Remarks on code availability)
The code appears well documented and the Readme file is clearly written. I appreciate the use of a demo case, which ran successfully.

Open Access This Peer Review File is licensed under a Creative Commons Attribution 4.0 International License, which permits use, sharing, adaptation, distribution and reproduction in any medium or format, as long as you give appropriate credit to the original author(s) and the source, provide a link to the Creative Commons license, and indicate if changes were made.
In cases where reviewers are anonymous, credit should be given to 'Anonymous Referee' and the source.
The images or other third party material in this Peer Review File are included in the article's Creative Commons license, unless indicated otherwise in a credit line to the material. If material is not included in the article's Creative Commons license and your intended use is not permitted by statutory regulation or exceeds the permitted use, you will need to obtain permission directly from the copyright holder.
To view a copy of this license, visit https://creativecommons.org/licenses/by/4.0/

Response Letter for manuscript NCOMMS-26-00940

We thank the reviewers for the careful evaluation of our manuscript and for their helpful feedback. Our replies to all reviewer comments can be found below in blue text color. The revised manuscript as well as a version with tracked changes compared to the original draft can be found as separate pdf files.

Following the suggestion of reviewers #1 (comment #1) and reviewer #2 (comment #2), we adapted the definition of the Amerasian Arctic domain (to also include the Bering Sea) as well as the time period for the “new Arctic” (changed from 2000-2024 to 2004-2024). Thus, the results depicted in most manuscript figures and tables have slightly changed compared to the original manuscript draft. The key conclusions of the manuscript did not change with these adaptions.

REVIEWER #1:

General Comments:

This study investigates the impact of serial cyclone clusters on Arctic sea-ice concentration (SIC), in comparison to solitary cyclones. Cyclone clusters produce a SIC reduction that is about twice as strong and lasts approximately 2.5 times longer than that of solitary cyclones. The SIC response has intensified from 1979–1999 to 2000–2024, indicating a changing relationship with background ice conditions beyond simple SIC decline.

Moreover, the impacts exhibit clear regional differences between the Eurasian and Amerasian sectors, highlighting spatial heterogeneity in cyclone–sea ice interactions. This is a novel attempt and presents an interesting analysis. However, several issues need to be addressed, including the choice of regions, comparisons across different periods, the influence of ocean heat transport and decadal variability, and the role of sea-ice thickness. In its current form, the study is not yet suitable for publication.

We thank reviewer #1 for the constructive feedback, which helped us to further improve our manuscript. Our responses to the individual comments can be found below.

Major Comments:

1. Choice of regions

Although this study focuses on the Arctic Ocean, the comparison between the Eurasian and Amerasian sectors is not equivalent. The Eurasian sector (Fig. 1a) always includes the ice edge and open-ocean areas (e.g., the Barents and Norwegian Seas) and is geographically well positioned to capture the Icelandic Low, particularly in winter. In contrast, the Amerasian sector (Fig. 1d) is almost entirely covered by sea ice during winter and is located far north of the main center of cyclone activity associated with the Aleutian Low.

If regions extending to the ice edge are to be considered, the Bering Sea and the Sea of Okhotsk should be included, where strong responses are in fact noted in the manuscript (line 111, Fig. 2c). A similar argument applies to the Labrador Sea. Therefore, in order to enable a fair seasonal

comparison of regions in this study, it is necessary to redefine the domains with consideration of the southern boundary of sea ice. At a minimum, an Amerasian sector encompassing the Bering and Okhotsk Seas, as well as a Canadian Archipelago sector encompassing the Labrador Sea, should be introduced.

Thank you for pointing us to this inconsistency in the domain definition between the Eurasian and Amerasian Arctic. We followed your advice and included the Bering Sea into the Amerasian Arctic, so that the cold season ice edge in the Pacific sector of the Arctic Ocean is included. However, we decided not to include the Sea of Okhotsk, as its sea-ice zone is separated from the rest of the study region by a landmass. In this respect, including the Sea of Okhotsk would be similar to including the Baltic Sea as part of the Eurasian Arctic. We further introduced a new, Canadian Archipelago sector encompassing the Labrador Sea, as suggested. The domains can be found in panels “a” and “d” of the new Figure 1:

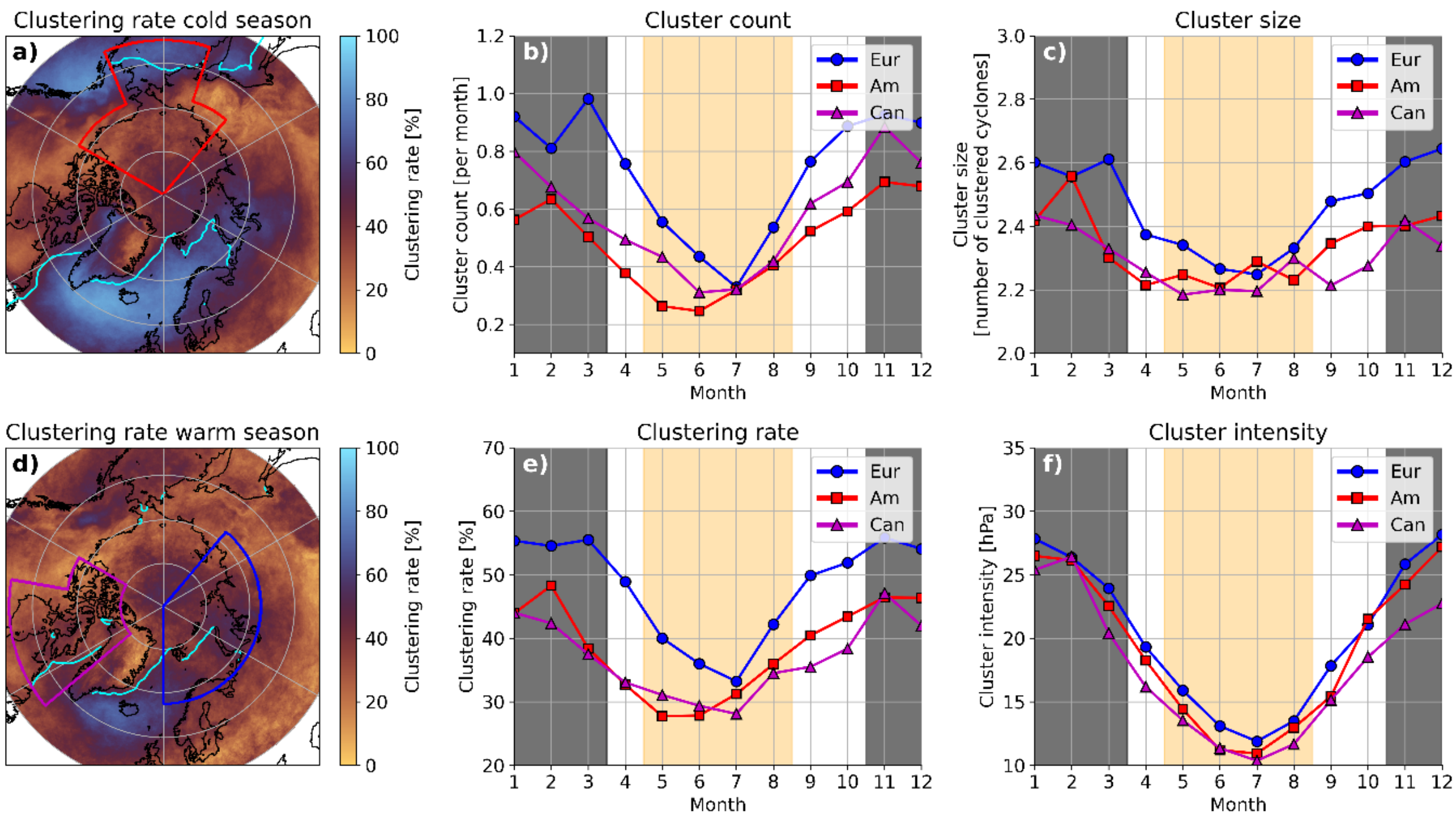

Figure 1: Occurrence and properties of cyclone clusters in the Arctic. Panels a and d indicate clustering rate (share of all cyclones which occur within clusters) for the cold season (NDJFM, a) and warm season (MJJA, d) for the years 2004-2024, and the light blue lines indicate the average position of sea-ice edge (15\% mean SIC contour). The remaining panels indicate the annual cycle of cyclone absolute cluster count (b), clustering rate (e), cluster size (c) and cluster intensity (f) for the Eurasian Arctic ("Eur", blue domain in panel d), the Amerasian Arctic ("Am", red domain in panel a) and the Canadian Archipelago sector ("Can", purple domain in panel d). Gray and yellow shadings highlight months in the cold and warm seasons, respectively.

The revised discussion of Figure 1 is found at lines 79-103 of the revised manuscript and reads:

“Within the Arctic, there are pronounced regional and seasonal variations in cyclone cluster occurrence (Fig. 1a,d). In alignment with previous studies[42], we find that cyclone clustering occurs frequently in the exit regions of the North Atlantic storm track in the cold season. These relatively high clustering rates of up to 70% extend further into the Atlantic marginal seas of the Arctic Ocean. Similarly high values are found in the Pacific sector of the Arctic including the Gulf of Alaska, the Bering Sea and the Sea of Okhotsk. In the central Arctic Ocean, the cold season clustering rate is

around 50%, and in the Beaufort, Chukchi and East Siberian Seas, most cyclones occur separately from each other. In the warm season, clustering rates are substantially lower along the Atlantic and Pacific storm tracks compared to the cold season. As a consequence, the regional maximum of Arctic warm season clustering rate is found in the Kara Sea and the central Arctic Ocean and amounts to approximately 50%. Throughout basically the entire study domain, seasonal clustering rates did not change significantly between the periods 1979-1999 and 2004-2024 (Supplementary Fig. 2).

Next, we divide the sea-ice covered regions of the Arctic into subdomains and analyze the annual cycle of cluster occurrence and properties. We define an Eurasian and an Amerasian sector of the Arctic Ocean following a previous study[41], but in this study extend the Amerasian Arctic southward to include the cold season ice edge located in the Bering Sea. We further introduce an additional Canadian Archipelago sector. All three domains are outlined in Figure 1.

The occurrence frequency of cyclone clusters exhibits a clear minimum in July for the Eurasian Arctic in both absolute counts and clustering rate (Figure 1b,e). In contrast, in the Amerasian Arctic, the minimum cyclone cluster occurrence is already reached in May to June. Hereby, the magnitude of the annual cycle is larger in the Eurasian Arctic than in the Amerasian Arctic and the Canadian Archipelago. This applies also for the mean cluster size (Figure 1c), defined as the number of cyclones per cluster. Despite the differences in terms of cluster occurrence, the annual cycle of cluster intensity (mean intensity of all clustered cyclones) is very similar for all three regions (Figure 1f), except for a slightly lower intensity in the Canadian Archipelago from October through December. As a consequence, the annual cycles of cluster occurrence and cluster intensity are shifted in time in the Amerasian Arctic (occurrence minimum in May-June, intensity minimum in June-July). While monthly cluster occurrence and properties are similar for 2004-2024 and 1979-1999 in the Eurasian Arctic, some deviations are found for the Amerasian Arctic, including a more pronounced yearly minimum in the clustering rate (and cluster size) in June for 1979-1999 than for the more recent period (Supplementary Fig. 2)."

Furthermore, table 1 was revised to include the regional mean SIC impact of cyclones for the revised Amerasian Arctic domain and the new Canadian Archipelago sector:

		**Magnitude**		**Duration**	
		**Solitary**	**Cluster**	**Solitary**	**Cluster**
**Cold season**	**Eurasian**	-1.8 %	-3.6 % (2.0)	2.4 d	6.3 d (2.6)
	**Amerasian**	-0.4 %	-2.7 % (6.8)	2.0 d	5.2 d (2.6)
	**Canadian**	-0.8 %	-1.8 % (2.3)	1.9 d	5.2 d (2.7)
	**Arctic**	-1.0 %	-2.1 % (2.1)	2.1 d	5.5 d (2.6)
**Warm season**	**Eurasian**	-2.4 %	-3.7 % (1.5)	2.1 d	5.4 d (2.6)
	**Amerasian**	-1.9 %	-3.2 % (1.7)	2.0 d	5.2 d (2.6)
	**Canadian**	-1.9 %	-2.3 % (1.2)	1.8 d	4.2 d (2.3)
	**Arctic**	-2.0 %	-3.1 % (1.6)	2.0 d	5.1 d (2.6)

Table 1: Domain-averaged sea-ice impact and duration. Magnitude and duration of SIC-impact during solitary cyclones and cyclone clusters for different seasons and regions. "Arctic" refers to entire domain north of 70 degrees north. Numbers in parenthesis indicate amplification factor between clusters and solitary cyclones.

Due to the southward extension of the Amerasian Arctic domain, a particularly strong intensification of cyclone cluster impacts compared to solitary cyclones is now found for this domain in the cold season, which is discussed at lines 113-117 of the revised manuscript:

"Averaged over the entire Arctic Ocean (north of 70°N), the cyclone cluster impact on SIC is 2.1 times stronger and lasts 2.6 times longer than for solitary cyclones (Table 1). Similar values are found for the Eurasian Arctic and the Canadian Archipelago sector, while in the Amerasian Arctic, the amplification of cyclone cluster impacts amounts to 6.8. This remarkably strong signal is driven by particularly intense SIC-reductions due to cyclone clusters in the Bering Sea (Fig. 2)."

References:

- 41. Finocchio, P. M., Doyle, J. D. & Stern, D. P. Accelerated sea ice loss from late summer cyclones in the new arctic. J. Clim. 35, 7751–7769 (2022).
- 42. Dacre, H. F. & Pinto, J. G. Serial clustering of extratropical cyclones: a review of where, when and why it occurs. npj Clim. Atmospheric Sci. 3, 48 (2020).

2. Choice of periods
The manuscript compares two periods, 1979–1999 and 2000–2024. A major difference between these periods is the transition of the Atlantic Multidecadal Oscillation (AMO) from a negative to a positive phase (e.g., Dima et al., 2025, https://doi.org/ 10.1038/s41612-025-00960-x). During the positive AMO phase (2000–2024), the Icelandic Low is expected to be stronger (although it is unclear whether this directly increases the number of cyclone clusters), and, at least, ocean heat transport (OHT) toward the Arctic—favorable for suppressing sea-ice growth—should be enhanced. Therefore, consideration of the AMO appears essential, particularly for the discussion of winter conditions in the Eurasian sector.

Alternatively, if the analysis is based on climate regimes, one could classify the Eurasian sector by AMO phase, while the Amerasian sector might be better categorized by the Pacific Decadal Oscillation (PDO). The PDO has transitioned from a positive phase in the 1980s toward a more negative phase in recent decades. In contrast to the Eurasian sector, this suggests conditions that are generally less favorable for sea-ice reduction (although PDO+ can also be associated with sea-ice loss in some cases).

In other words, a direct comparison between the Eurasian and Amerasian sectors may not be appropriate even before considering whether cyclones are solitary or clustered, because the background states of ocean heat transport—and thus the sea-ice growth environment—are fundamentally different. This raises the question of whether a simple period-based comparison is suitable, or whether an analysis centered on large-scale oceanic variability (e.g., AMO+ vs. PDO+, AMO- vs. PDO-) would yield different conclusions.

We thank reviewer #1 for pointing us to this interesting topic. We agree that it would be exciting to follow up on potential links between large-scale oceanic variability and regional cyclone (cluster) impacts on Arctic sea ice in more detail, but consider this to be a topic of a separate paper or even papers. To us, the primary result of this manuscript is the greater impact of serial cyclone clusters compared to solitary cyclones throughout the Arctic, while the inter-basin differences are a

secondary result. Thus, including additional analysis of contributions from large-scale oscillations to these inter-basin differences would make the paper more complex and harder to follow, while contributing only to one of the subordinate research questions.

To nonetheless address the potential relevance of large-scale oceanic variability for the presented findings, we added respective text and literature references:

Lines 119 – 121: ”Further, the Eurasian and Amerasian sectors of the Arctic (the latter including the Bering Sea) are characterized by different background states of ocean heat transport and thus different sea-ice growth/melt environments, related to the Atlantic Multidecadal Oscillation (AMO), on the one hand, and the Pacific Decadal Oscillation (PDO), on the other hand. A potential contribution of such large-scale oscillations to the inter-basin differences should thus be kept in mind when interpreting the results.”

Lines 286 – 290: ”Another major difference between the Old and New Arctic periods is the transition of the Atlantic Multidecadal Oscillation (AMO) from a negative to a positive phase[57]. As variations in large-scale oceanic variability such as the AMO or the Pacific decadal oscillation (PDO) control ocean heat transport into the Arctic and thus the sea-ice growth/melt environments in which the impacts of cyclone clusters take place, an analysis centered on this large-scale oceanic variability could reveal additional insights.”

References:

- 57. Dima, M., Lohmann, G., Nichita, D.-R., Knorr, G. & Scholz, P. Structural stability changes of the atlantic meridional overturning circulation. npj Clim. Atmospheric Sci. 8, 73 (2025).

3. Treatment of cyclones across the Eurasian and Amerasian sectors

Consider the Great Arctic Cyclone mentioned in the Introduction (lines 28–30). This cyclone traverses from the Eurasian sector to the Amerasian sector as defined in this study. In such cases, clarification is needed on how these cyclones are classified—i.e., which sector they are assigned to. Furthermore, according to Yamazaki et al. (2015, 10.1002/2014JD022925), this event developed through the coupling of a surface cyclone over the Beaufort Sea with an upper-tropospheric Polar Vortex advected from the Eurasian sector. From this dynamical perspective, it is unclear whether such a case should be categorized as a solitary cyclone or as part of a cluster.

Since the cyclone tracking in this study is based on sea level pressure (SLP), it relies on detecting and following surface cyclones. However, to fully capture the dynamical structure of such events—including vertical coupling processes—it may be necessary to also consider fields at 500 hPa or 300 hPa. This limitation of the analysis should be explicitly addressed in the Discussion. In addition, it would be helpful to clarify how the Great Arctic Cyclone is categorized within the framework of this study (e.g., in Fig. 4). Providing a concrete example would also strengthen the connection to the Introduction.

Thank you for making us aware of the lack of clarity regarding the regional break-down of the cyclone impacts on sea ice. Our analysis of the SIC-response to cyclones is purely Eulerian. That means, the SIC-response is calculated separately at each ice-covered grid-cell of the analysis domain, whenever the grid-cell is overpassed by a cyclone. Hereby, each individual cyclone will influence several ice-covered grid-cells throughout its lifetime as it moves through the Arctic. This also means that some

cyclones - such as the Great Arctic cyclone mentioned in the Introduction - will affect grid-cells in both the Eurasian Arctic and the Amerasian Arctic. To clarify this, we added more details on how the regional mean SIC-impacts are calculated in the Methods section at lines 361-364:

"Regional mean impacts on SIC (e.g. in Table 1) are calculated as spatial averages over all (ice-covered) grid-cells of the respective domains. Thus, cyclones that pass over grid-cells in not just one but multiple domains during their lifetime contribute to the regional mean SIC impacts in multiple domains. The same applies when regional mean SIC impacts are calculated for different categories of initial sea-ice conditions."

Since in the framework of our study, the Great Arctic cyclone cannot clearly be associated with only one specific domain, we prefer not to include this example in the discussion of Figure 4, even though we agree that it would have been nice to reconnect to the Introduction here.

We further acknowledge that our SLP-based cyclone tracking has limitations in capturing vertical coupling processes between lower and upper troposphere, and added according text at lines 290-292:

"It should further be noted that our differentiation between cyclone clusters and solitary cyclones is solely based on sea-level pressure fields. Consequently, vertical coupling processes between surface cyclones and upper-level atmospheric features – which, for example, played a key role for the Great Arctic Cyclone in 2012[58] - are not captured in our analysis."

References:

- 58. Yamazaki, A., Inoue, J., Dethloff, K., Maturilli, M. & König-Langlo, G. Impact of radiosonde observations on forecasting summertime arctic cyclone formation. J. Geophys. Res. Atmospheres 120, 3249–3273 (2015).

4. Sea-ice thickness (SIT)

The role of SIT is briefly mentioned in the Discussion (lines 274–278), but it is not sufficiently explored. There is clear potential to incorporate SIT information from ocean reanalyses, climate model outputs, or satellite-based observations. The dynamical response of sea ice to wind forcing strongly depends on SIT and is therefore crucial for interpreting the results of this study. In addition, from the perspective of OHT, wind-driven processes that suppress sea-ice growth are also closely linked to SIT. Without adequately addressing these aspects, the mechanisms underlying the reported SIC changes remain unclear. In this sense, the manuscript provides an incomplete interpretation, as the lack of a thorough discussion of SIT limits the ability to explain the physical processes behind the presented results.

Thank you for bringing up the very relevant topic of the role of sea-ice thickness. Including sea-ice thickness data into our analysis is challenging, since satellite-based products are too limited in their temporal extent and resolution (only available as weekly averages and from 2010 onwards) and relating SIT information from climate models (or ocean reanalysis) to the SIC response to cyclones obtained from satellite data (and ERA5) would likely introduce inconsistencies into the analysis.

We agree that the interpretation of our results regarding SIC changes during cyclone clusters would be strengthened, if a potential relation to SIT could be directly explored. We therefore added additional analysis based on the GLORYS12 Version 1 ocean reanalysis (GLORYS12 hereafter) into the Supplement. To minimize potential inconsistencies arising from the inclusion of an additional

dataset, we first carefully evaluated whether GLORYS12 is capable to reproduce a SIC response to cyclone clusters comparable to that obtained from observations/ERA5. Since GLORYS12 is only available from 1993 onwards, we could only conduct this additional analysis for the "New Arctic" period (2004-2024).

The new findings on the relevance of SIT for the SIC response to cyclone clusters are discussed at lines 275 – 279 of the revised manuscript:

"Following sea-ice dynamics theory, this could be related to the recent strong decline in Arctic SIT detected utilizing PIOMAS[56] reanalysis data (Supplementary Fig. 4). This SIT decline should lower the ice strength and thus facilitate atmospheric impacts on sea ice[49]. Utilizing additional SIT data from the GLORYS12[57] ocean reanalysis (see Methods section) for the new Arctic period (2004-2024), we indeed find that the strength of cyclone cluster impacts on SIC increases as SIT decreases below 1 m (Supplementary Fig. 5). This is particularly pronounced for the Eurasian cold season."

Below, the new Supplementary Figures 5 & 6 can be found, which show the dependence of the SIC-response to cyclone clusters on the initial SIT based on GLORYS12 (Supplementary Fig. 5) and the evaluation of the SIC-response to cyclone clusters in GLORYS12 with the ERA5-based response (Supplementary Fig. 6).

Supplementary Fig. 5:

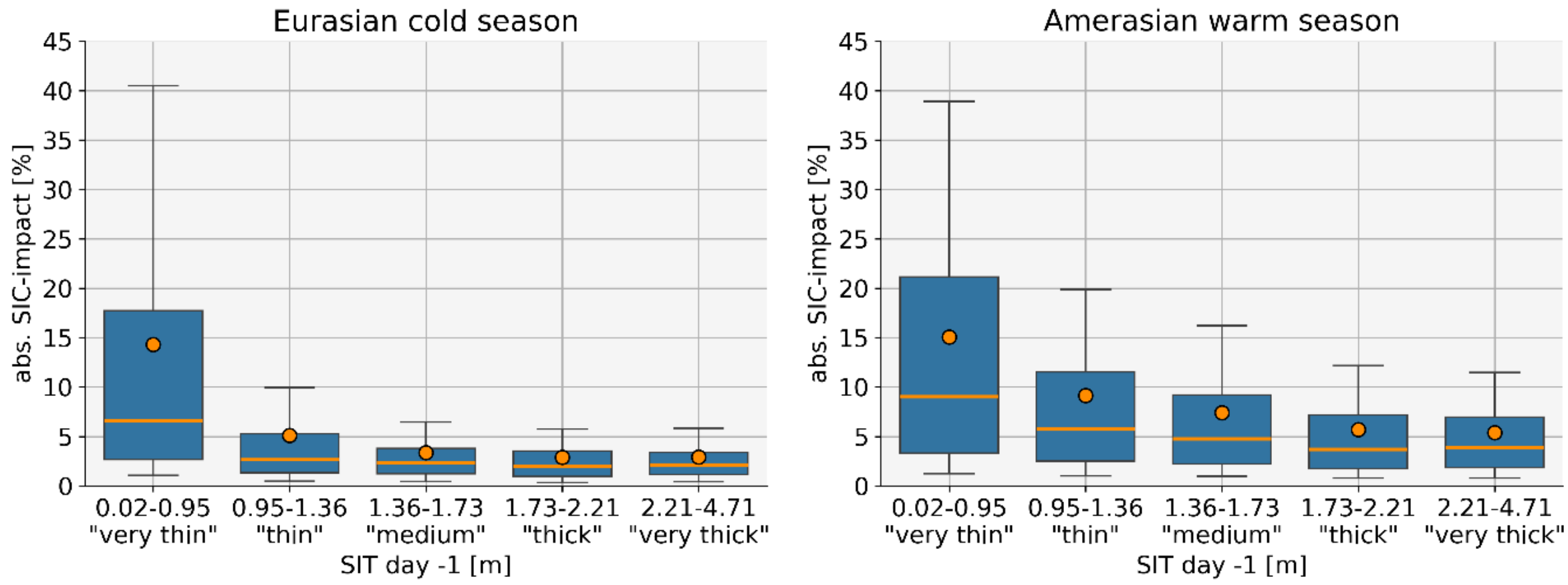

Supplementary Fig. 5: Influence of sea-ice thickness on sea-ice concentration response to cyclone clusters. Box-Whisker Plot of absolute sea-ice concentration (SIC) changes during cyclone clusters for different categories of initial sea-ice thickness (SIT) one day before the cyclone cluster arrival at an ice-covered grid-cell. Dots indicate the mean value, horizontal lines the median value, and box (whisker) edges indicate the 25th/75th (10th/90th) percentiles. SIT-bins are defined by the 0th, 20th, 40th, 60th, 80th and 100th percentile of the pan-arctic SIT distribution. The left panel shows results for the Eurasian Arctic during the cold season (NDJFM), while the right panel shows results for the Amerasian Arctic during the warm season (MJJA), both for the New Arctic period 2004-2024. SIC and SIT data originate from the GLORYS12 version 1 ocean reanalysis[56].

Supplementary Fig. 6:

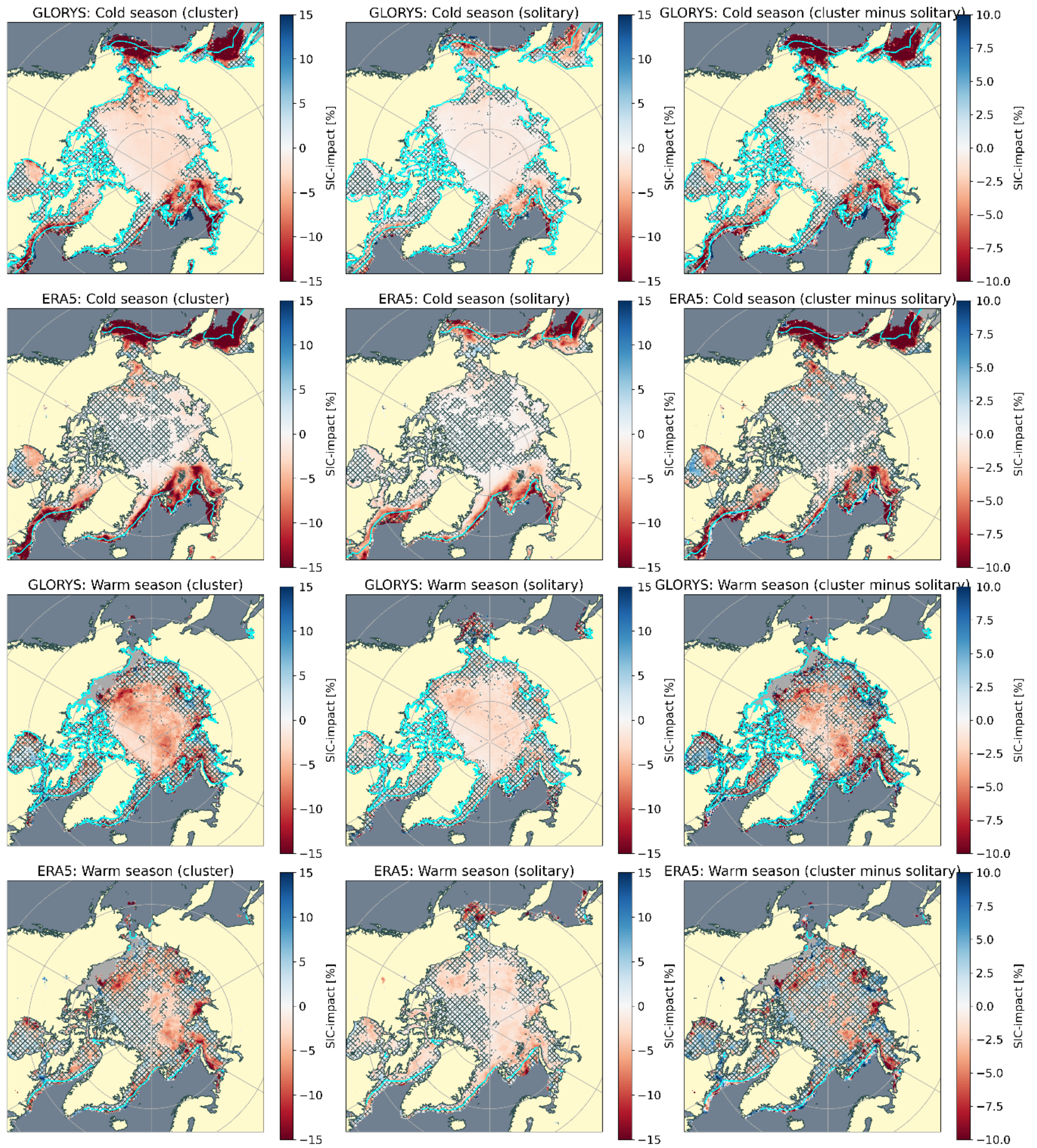

Supplementary Figure 6: Evaluation of Impact of cyclone clusters and solitary cyclones on sea ice in GLORYS12. Mean SIC-change (relative to non-cyclone reference) during cyclone clusters (left column), during solitary cyclones (middle column), and the difference between clusters and solitary cyclones (cluster minus solitary, right column). The first row indicates the cold season (NDJFM) and GLORYS12 data[57], the second row indicates the cold season (NDJFM) and ERA5 data[46], the third row indicates the warm season (MJJA) and GLORYS12 data, and the bottom row indicates the warm season (MJJA) and ERA5 data. All panels show New Arctic period from 2004-2024. Light blue line indicates position of sea-ice edge (15% mean SIC contour). Hatching indicates grid-cells, where the signal is not statistically significant. Ice-covered grid cells are color shaded only if they have at least 5 cyclone (cluster) samples.

We further added a new paragraph to the methods section (at lines 319-325 under "Data"), where we briefly describe the utilized sea-ice thickness datasets:

"Further, sea-ice thickness (SIT) data from the Pan-Arctic Ice Ocean Modeling and Assimilation System PIOMAS[56] as well as SIT and SIC data from the CMEMS global ocean eddy-resolving reanalysis GLORYS12[57] version 1 (hereafter GLORYS12) are utilized. PIOMAS data are used to assess long-term changes in SIT between the Old Arctic and New Arctic periods (1979-1999 and 2004-2024, respectively). GLORYS12, which is only available from 1993 onward but is more advanced in terms of model physics, resolution, and data assimilation, is used to analyze the role of sea-ice thickness in modulating cyclone-induced SIC changes during the New Arctic period. The qualitative similarity in the SIC response to cyclones in ERA5/satellite data and in GLORYS12 demonstrates that GLORYS12 is suitable for this purpose (Supplementary Fig. 6)."

References:

- 46. Hersbach, H. et al. The era5 global reanalysis. Q. J. Royal Meteorol. Soc. 146, 1999–2049 (2020).
- 49. Hibler, W. D. A dynamic thermodynamic sea ice model. J. Phys. Oceanogr. 9, 815–846 (1979).
- 56. Zhang, J. & Rothrock, D. A. Modeling global sea ice with a thickness and enthalpy distribution model in generalized curvilinear coordinates. Mon. Weather. Rev. 131, 845–861 (2003).
- 57. Jean-Michel, L. et al. The copernicus global 1/12° oceanic and sea ice glorys12 reanalysis. Front. Earth Sci. Volume 9 - 2021 (2021).

Minor comments:
1. Title: It would be more accurate to refer to *Arctic sea-ice extent* rather than *Arctic sea ice* in the title.

We thank you for the suggestion, but prefer to keep the title unchanged, as the term *Arctic sea-ice extent* is often understood as a single number indicating the panarctic sea-ice area, while our study mostly focusses on regional impacts on sea-ice concentration fields.

2. Figs. 5 & 6: They are hard to understand.

Thank you for pointing us to this issue. We acknowledge that Figures 5 & 6 contain a lot of information and are thus quite complex, but we are convinced that they contribute significantly to our research objectives. To make the interpretation of the figures easier to follow, we have revised the wording of the manuscript text in lines 193–195 where Figure 5 is first introduced:

"The impact of cyclone clusters on SIC is expected to depend on the characteristics of the sea ice they overpass. This dependence is illustrated in Figs. 5 and 6, which depict the distribution of SIC changes during cyclone clusters for different initial SIC conditions prior to the cyclone passage. Results are presented separately for 2004-2024 (pink-framed boxes) and 1979-1999 (cyan-framed boxes."

Reviewer #2:

Summary and Key Results: This manuscript aims to quantify the impact of cyclone clusters to very rapid sea ice loss events in the Arctic. Cyclone clusters are when there are several cyclones that follow in near spatial and temporal proximity to each other. The analysis is conducted using ERA5 atmospheric reanalysis, ERA5 sea ice concentration (SIC), and a freely available cyclone tracking algorithm. The authors find that cyclone clusters contribute to significantly more loss in SIC over a longer amount of time compared to single (solitary) cyclone events. They also find that the losses in SIC have increased in recent times.

Overall, this manuscript provides a convincing case that cyclone clusters have a larger impact on SIC decreases than single cyclones. My expertise lies mostly within atmospheric dynamics, yet I struggled to understand the section "Role of initial SIC prior to cyclone passage." In particular, I find the last part of the analysis confusing (the decomposition of the changed SIC impact), and it is not clear what it ends up adding to the conclusions. Thus the presentation of this section could need additional work, given that Nature Communications aims to reach a broad, global audience of professional researchers, academics, and specialists across all natural sciences. There are some other issues that need addessing, particularly in the methods, with some other issues in clarity needed. Once these issues have been addressed, I think this article will make a excellent contribution to Nature Communications.

We thank reviewer #2 for the positive feedback and for pointing us to issues which need further improvement. Our responses to the individual comments can be found below.

To address your comment that the idea behind the decomposition of the changed SIC impact does not become fully clear, we added an additional paragraph at the start of this section to motivate the analysis (at lines 224-229 of the revised manuscript):

"It has been hypothesized that intensification of cyclone-induced SIC changes in the Arctic in recent years is primarily driven by the decline in mean SIC and the associated reduction in sea-ice strength[50]. In contrast, our results indicate that cyclone (cluster) impacts on SIC have intensified in the New Arctic period even within fixed SIC conditions (Figs. 5 and 6). This finding suggests that also factors other than the ongoing SIC-decline contribute to the observed intensification. To clarify this, we first examine how the composition of the Arctic sea-ice cover with respect to the previously defined SIC categories has changed between the Old and New Arctic periods. We then explicitly quantify the extent to which these shifts in SIC conditions contribute to the strengthening of cyclone-cluster impacts."

References:

- 50. Aue, L. & Rinke, A. Cyclone impacts on sea ice concentration in the atlantic arctic ocean: Annual cycle and recent changes. Geophys. Res. Lett. 50, e2023GL104657 (2023).

Significance: It is reasonable to expect that a series of cyclones will lead to a larger net reduction of sea ice than single cyclone events. This study quantifies this expectation, and furthermore shows that these impacts are increasing in the New Arctic. However, I think the societal relevance could be better stressed. For example, cyclones, reductions in sea ice, thawing permafrost, and increasing river flows are leading to notable increases in hazardous weather events in and around the Arctic region (e.g., Valkonen et al. 2021; Zhang et al. 2023). Coastal regions are now facing substantial erosion and flooding, particularly when impacted by cyclones. Away from coastal regions, reduced

sea ice increasingly reveals oil reserves within the Arctic Ocean, motivating bordering countries to extend their sovereignty claims in international waters (e.g. Gerhardt et al. 2010; Carlson et al. 2013) and has led to a dramatic increase in shipping in the Arctic due to the increased accessibility and shorter shipping routes, particularly during the summer (e.g., Stephenson et al. 2013; Stephenson and Smith 2015).

Thank you for pointing us to these helpful references. We included some of them in the Introduction section at lines 34-38 in order to better stress the societal relevance of our study:

"From a more practical perspective, the strong ice drift and enhanced wave activity during cyclone-related storms can also affect maritime economic activities in the Arctic[15, 16], which have expanded considerably in recent years due to the increased accessibility and shorter shipping routes[17, 18]. At the same time, Arctic coastal communities are increasingly exposed to hazardous weather events due to intensified cyclone activity[19, 20], while reductions in sea-ice cover and thawing permafrost have increased the vulnerability of coastal environments to storm-related impacts[21]."

References:

- 15. Waseda, T., Nose, T., Kodaira, T., Sasmal, K. &Webb, A. Climatic trends of extreme wave events caused by arctic cyclones in the western arctic ocean. Polar Sci. 27 (2021).
- 16. Inoue, J. Review of forecast skills for weather and sea ice in supporting arctic navigation. Polar Sci. 27, 100523 (2021).
- 17. Stephenson, S. R., Smith, L. C., Brigham, L. W. & Agnew, J. A. Projected 21st-century changes to arctic marine access. Clim. Chang. 118, 885–899 (2013).
- 18. Stephenson, S. R. & Smith, L. C. Influence of climate model variability on projected arctic shipping futures. Earth's Futur. 3, 331–343 (2015).
- 19. Zhang, X. et al. Arctic cyclones have become more intense and longer-lived over the past seven decades. Commun. Earth Environ. 4, 348 (2023).
- 20. Valkonen, E., Cassano, J. & Cassano, E. Arctic cyclones and their interactions with the declining sea ice: A recent climatology. J. Geophys. Res. Atmospheres 126, e2020JD034366 (2021).
- 21. Henke, M., Miesse, T., de Lima, A. d. S., Ferreira, C. M. & Ravens, T. M. Increasing coastal exposure to extreme wave events in the alaskan arctic as the open water season expands. Commun. Earth Environ. 5, 165 (2024).

Data and methodology, analytic approach, and suggested improvements:

1) PIOMAS sea ice thickness is used in the sea ice thickness analysis, but it is not mentioned or described in the data and methods section.

Thank you for capturing this. We added a new paragraph to the methods section (at lines 319-325 under "Data"), where we briefly describe the utilized sea-ice thickness datasets:

"Further, sea-ice thickness (SIT) data from the Pan-Arctic Ice Ocean Modeling and Assimilation System PIOMAS[56] as well as SIT and SIC data from the CMEMS global ocean eddy-resolving reanalysis GLORYS12[57] version 1 (hereafter GLORYS12) are utilized. PIOMAS data are used to assess long-term changes in SIT between the Old Arctic and New Arctic periods (1979-1999 and 2004-2024, respectively). GLORYS12, which is only available from 1993 onward but is more advanced in terms of model physics, resolution, and data assimilation, is used to analyze the role of sea-ice thickness in

modulating cyclone-induced SIC changes during the New Arctic period. The qualitative similarity in the SIC response to cyclones in ERA5/satellite data and in GLORYS12 demonstrates that GLORYS12 is suitable for this purpose (Supplementary Fig. 6).”

References:

- 56. Zhang, J. & Rothrock, D. A. Modeling global sea ice with a thickness and enthalpy distribution model in generalized curvilinear coordinates. Mon. Weather. Rev. 131, 845–861 (2003).
- 57. Jean-Michel, L. et al. The copernicus global 1/12° oceanic and sea ice glorys12 reanalysis. Front. Earth Sci. Volume 9 - 2021 (2021).

2) The authors separate data into two periods: one corresponding to the so-called “Old Arctic” (1979–1999) and the other the “New Arctic” (2000–2024). This idea is reasonable, except I wonder why the two periods don’t have the same number of years. This makes it harder to compare because the “New Arctic” has a much larger sample size. Seems like it would be most simple and fair to just have the “New Arctic” start at 2004. This would likely make the authors’ results stronger, since 2007 was the first drastic reduction in year-to-year sea ice extent.

Thank you for this reasonable suggestion. We changed the new Arctic period definition to 2004-2024, so that the equal sample size makes the comparison to the old Arctic period fairer, and updated the manuscript figures and tables. There are only minor changes in the basic results of the study, indicating that they are not overly sensitive to the choice of years for defining the new vs. old Arctic. Where the results changed, they mostly indicate a stronger/more intensified impact of cyclones for the revised new Arctic definition, as expected.

3) The analysis seems incomplete when choosing not to include April, September, and October. The text does not provide very clear reasons as to how the authors decided how to exclude certain months. For example, they state the October is excluded because it “differs substantially” from the following months. But what does “substantial” mean (line 318)? Is there a number being used to support this conclusion? I think the authors are ultimately looking to divide the sample into two periods within each year: One where sea ice is thicker and extensive with relatively low spatial variability (limited mainly to the MIZs), and the other where sea ice is thinner and less extensive with relatively high spatial variability. Perhaps the combination of these specific factors is quantifiable. Or if the authors expect a different relationship between cyclones and sea ice, a “shoulder seasons” analysis could also be included.

Thank you for pointing out the lack of clarity here. We acknowledge that no quantitative measure has been used to decide on the monthly grouping. Instead, we chose the grouping to separate times of the year where the sea-ice concentration in the Arctic is usually (in the absence of cyclones) increasing (referred to as cold season) or decreasing (referred to as warm season). If both cases would be mixed, it would be hard to interpret cyclone anomalies in SIC changes, because the base state of the SIC-change would be unclear. We explain this strategy at lines 328-330 of the (revised) manuscript:

“The specific grouping of months is designed to differentiate between seasons when the SIC in the Arctic is usually increasing or decreasing due to sea-ice growth or melt (Supplementary Fig. 7). This

facilitates the interpretation of relative SIC-change signals for cyclones compared to non-cyclone conditions."

We acknowledge that our argument to exclude October from the cold season might be a bit unclear. Thus, we tried to clarify the respective wording at lines 330-332:

"October is not included in the cold season because, relative to the subsequent months, the mean sea-ice extent is still lower and the climatological increase in SIC (in the absence of cyclones) is pronounced stronger, specifically along the Siberian coast (Supplementary Fig. 7)."

Figure S7:

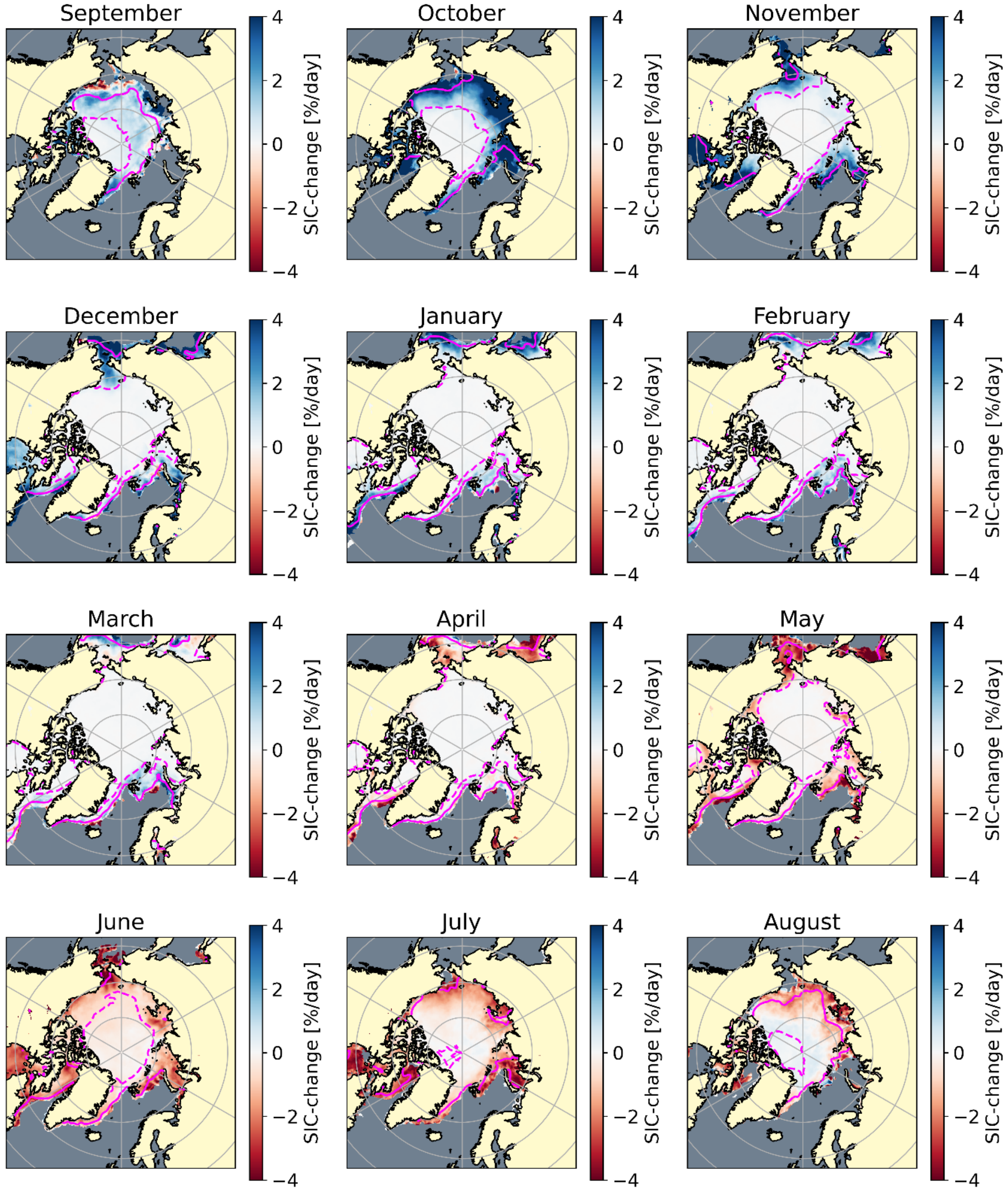

Fig. S7. Non-cyclone reference SIC-change. Non-cyclone reference SIC-change for each month of the years 2004-2024. Solid (dashed) magenta line indicates 15% (90%) mean SIC contour.

4) 83–84: What would make clustering rates be considered "similar" vs. being considered "not similar?" Is there a threshold? Or is it arbitrary?

Thank you for pointing out this unclarity. We added the explicit difference in seasonal clustering rates between Old and New Arctic to Supplementary Fig. 2, including a significance test (student's t-test on 95% confidence level), and adapted the corresponding text at lines 87-88 of the revised manuscript to be more specific: "Throughout basically the entire study domain, seasonal clustering rates did not change significantly between the periods 1979-1999 and 2004-2024 (Supplementary Fig. 2)."

5) 114: The word "generally" is not needed. The comparison is between -0.3% and -0.4%, which I doubt is quantfiably different.

Thank you for this comment. We agree that this difference was indeed too small to be considered meaningful. With the southward extension of the Amerasian Arctic domain (see general comment at start of response letter and revised Figure 1), the mean impact of cyclone clusters has substantially intensified for this domain in the cold season. The new values in Table 1 for solitary cyclones and cyclone clusters are -0.4% vs -2.7%. We revised the text at lines 113-117 accordingly:

"Averaged over the entire Arctic Ocean (north of 70°N), the cyclone cluster impact on SIC is 2.1 times stronger and lasts 2.6 times longer than for solitary cyclones (Table 1). Similar values are found for the Eurasian Arctic and the Canadian Archipelago sector, while in the Amerasian Arctic, the amplification of cyclone cluster impacts amounts to 6.8. This remarkably strong signal is driven by particularly intense SIC-reductions due to cyclone clusters in the Bering Sea (Fig. 2)."

Clarity and Context:

1) How are SLP "anomalies" defined (line 324)? Is it the anomaly with respect to a long-term climatology, or is it a spatial anomaly? If it is respect to a long-term climatology, what years are used? And is it daily, monthly, or seasonal averages that are being subtracted?

Thank you for pointing out the missing details in the description of the cyclone tracking procedure. We added the requested information at lines 338 – 341 of the revised manuscript:

"Cyclones are identified as negative SLP anomalies of at least -8 hPa strength (standard parameter setting of MOAAP). To calculate this SLP anomaly, the 6-hourly SLP field from ERA5 is first smoothed with a uniform square filter with a length of 100 km to remove small-scale noise and local orographic effects, and afterwards compared to a background SLP environment obtained by applying a uniform square filter with a side length of 3000 km and a temporal extent of 78 hours[47]."

References:

- 47. Prein, A. F., Mooney, P. A. & Done, J. M. The multi-scale interactions of atmospheric phenomenon in mean and extreme precipitation. Earth's Futur. 11, e2023EF003534 (2023).

2) What exactly is "cluster intensity?" Cyclone intensity is described on lines 332–334, but it's not obvious to me exactly how this applies to a cluster of cyclones.

Thank you for pointing out this lack of explanation. The intensity for a cluster of cyclones is calculated as the average over the intensities of all cyclones within the cluster. We added text at lines 350-351 of the revised manuscript to explain this:

"For cyclone clusters, this means that the cluster intensity is the average intensity over all cyclones within the cluster."

3) For both the Eurasian cold season (Figure 5) and Amerasian warm season (Figure 6) analyses, the authors perfrom a hypothesis test that changes in cyclone cluster trajectories between the "new" and "old" Arctic periods could be responsible for dynamically-driven SIC loss. Some more context is needed for how this test is expected to eliminate or support the possibility that the SIC losses are dynamically driven. In both cases, there is no statistical significance (though the authors explcitly point this out in the text for the former but not the latter). Thus the authors seem to be eliminating the possibility of dynamically-driven changes in SIC since there is no statistical significance. They then show evidence that SIT is decreasing between the two periods. But this would be dynamically-driven also (i.e., storms can more easily impact sea ice if the ice is thinner). Is this what the authors are trying to show? If so, it needs to be better explained. If not, perhaps this analysis is not necessary and could be removed or strongly condensed.

Thank you for making us aware that it is hard to follow our arguments regarding the changes in cyclone trajectories. We decided to follow your advice and removed the old supplementary figures 4 and 6 showing the (mostly not significant) changes in cyclone cluster trajectories as well as the respective discussion of the results.

Furthermore, we added a new figure to the supplement of the manuscript in order to directly quantify how the response of the SIC to cyclone clusters depends on the SIT prior to the cluster arrival, utilizing ocean reanalysis data (see also our reply to comment #4 of reviewer #1). These new findings are discussed at lines 275 – 279 of the revised manuscript:

"Following sea-ice dynamics theory, this could be related to the recent strong decline in Arctic SIT detected utilizing PIOMAS[56] reanalysis data (Supplementary Fig. 4). This SIT decline should lower the ice strength and thus facilitate atmospheric impacts on sea ice[49]. Utilizing additional SIT data from the GLORYS12[57] ocean reanalysis (see Methods section) for the new Arctic period (2004-2024), we indeed find that the strength of cyclone cluster impacts on SIC increases as SIT decreases below 1 m (Supplementary Fig. 5). This is particularly pronounced for the Eurasian cold season."

References:

- 49. Hibler, W. D. A dynamic thermodynamic sea ice model. J. Phys. Oceanogr. 9, 815–846 (1979).
- 56.
- 57. Jean-Michel, L. et al. The copernicus global 1/12° oceanic and sea ice glorys12 reanalysis. Front. Earth Sci. Volume 9 - 2021 (2021).

4) On lines 233–235, "all samples" should be changed to "all samples where there is SIC>15%."

Thank you for capturing this. We revised the wording according to your suggestion.

5) While ice-free summers will limit the impact that warm-season cyclones can have on sea ice in the future, won't this just shift the impacts into the shoulder seasons (lines 286–288)? There is no reason to expect anything to be different about the causes.

Thank you for pointing out the unclarity in the discussion of the respective literature reference. To account for your comment and free up space in the Discussion section to address other reviewer comments, we deleted the respective sentence.

Literature Review:

1) 28: To be consistent with Cavallo et al. (2025), the sea ice loss is not just extreme, but it is also very rapid (i.e., VRILEs).

Thank you for capturing this, we revised the wording at line 28 from "extreme" to "extreme and very rapid" as suggested.

2) The literature review considers "summer" and "winter" cyclones, but the analysis is for "warm" and "cold" season cyclones. Are the two meant to be equivalent? And what about the shoulder seasons? Or are the authors trying to divide the analysis into "warm" season and "non-warm" season?

Thank you for pointing out this inconsistency. We changed the wording in the introduction section from "summer" ("winter") to "warm season" ("cold season") to be consistent throughout the manuscript.

The grouping of months is chosen to separate times of the year where the sea-ice concentration in the Arctic is usually (in the absence of cyclones) increasing (cold season) or decreasing (warm season). The shoulder seasons are excluded, since sea-ice concentration change signals are rather unclear/mixed at that time of the year.

Typos and misspellings:

1) 17: season → seasons

Thank you for capturing this. Within the course of the manuscript revision, this part of the sentence was deleted, so that the typo is no longer included.

2) 309: 25km → 25 km

Thank you for capturing this. We revised the text accordingly.

3) 361–362: There should be a comma at the end of the equation labeled as equation (2), and "where" should not be indented on line 362.

Thank you for capturing this. We revised this part accordingly.

References

Carlson, J. D., C. Hubach, J. Long, K. Minteer, and S. Young, 2013: Scramble for the Arctic: Layered Sovereignty, UNCLOS, and Competing Maritime Territorial Claims. SAIS Review of International Affairs, 33 (2), 21–43.

Cavallo, S. M., M. C. Frank, and C. M. Bitz, 2025: Sea ice loss in association with Arctic cyclones. Communications Earth & Environment, 6 (1), 1–9, doi:10.1038/s43247-025-02022-9.

Gerhardt, H., P. E. Steinberg, J. Tasch, S. J. Fabiano, and R. Shields, 2010: Contested sovereignty in a changing Arctic. Annals of the Association of American Geographers, 100 (4), 992–1002.

Stephenson, S. R. and L. C. Smith, 2015: Influence of climate model variability on projected Arctic shipping futures. Earth's Future, 3 (11), 331–343.

Stephenson, S. R., L. C. Smith, L. W. Brigham, and J. A. Agnew, 2013: Projected 21st-century changes to Arctic marine access. Climatic Change, 118 (3-4), 885–899.

Valkonen, E., J. Cassano, and E. Cassano, 2021: Arctic cyclones and their interactions with the declining sea ice: A recent climatology. Journal of Geophysical Research: Atmospheres, 126 (12), e2020JD034 366, doi:10.1029/2020JD034366.

Zhang, X., H. Tang, J. Zhang, J. E.Walsh, E. L. Roesler, B. Hillman, T. J. Ballinger, and W.Weijer, 2023: Arctic cyclones have become more intense and longer-lived over the past seven decades. Communications Earth & Environment, 4 (1), 348.

Reviewer #3:

Summary:

This study quantifies the effect of Arctic cyclones that occur in series, addressing an important gap in the understanding of storm effects in the polar regions. Notable differences between warm and cold season impacts and storm effects in the “new” and “old” Arctic are presented. This paper assesses important variables including the occurrence, size, and intensity of cyclone clusters, as well as changes in the underlying sea ice concentration (SIC) field between time periods. Ultimately, the authors find the impacts of cyclone clusters to be greater than solitary storms and that there is an intensification of SIC-loss due to cyclone clusters from 1979-1999 to 2000-2024, which cannot solely be explained by the changing SIC distribution in the Arctic. I found the decomposition of SIC decline and intensification of cyclone impacts to be a very interesting novel result, which, (as the authors point out in the Discussion) opens new lines of research related to the effects of sea ice thickness and upper ocean mixing on sea ice changes in the Arctic. This work provides important context for other studies on Arctic cyclone impacts and sea ice decline, highlighting the significance of considering cyclone clustering on SIC.

The paper is clearly written, and the introduction provides thorough context with appropriate references for the results that follow. The figures are very effective, and the analysis presented here is generally well thought out with the results supporting the claims made. The data and methodology are also well described, and the work could be reproduced. I believe the research is noteworthy and deserving of publication in Nature Communications. I have a few small suggestions (listed below), which are relatively minor and should hopefully not require too much new analysis or significant changes to the manuscript.

We thank reviewer #3 for the constructive feedback which helped us to further improve our manuscript.

Major Comments:

My understanding of the methodology is that SIC differences are computed from one day before the first cyclone in a cluster to the last day of the final cyclone in the cluster. For the values in Table 1, it makes sense that the clusters have about 2.5 times the duration of solitary cyclones because there are more passing storms, however since the magnitude of SIC impact is only about twice as large as solitary cyclones, do clustered storms have a smaller effect per day on the ice than solitary storms? I would appreciate some clarification on the interpretation of these values.

Thank you for raising this important point. It is indeed true that cyclone clusters usually do not cause stronger SIC changes per day than solitary cyclones. This fits also to our finding that there are no strong differences in cyclone intensity between clustered and non-clustered cyclones (not shown). The main relevance of cyclone clustering is that the effects on SIC accumulate (which particularly in the cold season prevents a “recovery” of the sea-ice cover in between storms), so that stronger overall reductions in SIC occur. We tried to make this clearer in the discussion of Table 1 at lines 128-133:

“With approximately two-times (1.5-times) larger SIC-impact magnitudes in the cold (warm) season compared to 2.6-times longer event durations, our results suggests that the amplification of

cumulative SIC reductions for cyclone clusters is primarily driven by the prolonged cyclonic wind forcing, rather than by consistently stronger daily SIC changes. The only exception is the Amerasian Arctic during the cold season, where a 6.8-fold increase in SIC impact magnitude combined with a 2.6-fold increase in event duration demonstrates that there is a stronger cumulative SIC-reduction per unit time for cyclone clusters compared to solitary cyclones."

Related to this, I wonder if it's possible to include in Table 1 the difference in SIC impacts between the first storm of the cluster and the following 1-2 cyclones to identify any "preconditioning" the first storm might do to the ice (where the later storm might have a larger or smaller impact than the previous storm). I recognize this is an additional analysis that may not fit within this paper, but just an idea to better compare cyclone impacts on similar (one storm) timescales.

Thank you for this very interesting suggestion. We think that the idea to analyze preconditioning effects within a cyclone cluster is very exciting, but also requires careful methodological consideration. Specifically, cyclones within a cluster will potentially have different intensities, sizes and durations, which could complicate the interpretation of the results, and it might be difficult to define a clear cutoff between subsequent cyclones, as cyclone impacts on SIC can last for several days after a cyclones' passage under regular (non-clustered) conditions. We therefore believe it would be more promising to address this topic in a separate study, possibly involving the analysis of model results, and focus on the amplified overall impact of clusters vs solitary cyclones for now.

Line-by-Line Comments:

17-18, 112, 119-120, 260: Along with my general comment above, the end of these sentences seems to imply that the cyclones impacts are twice as large for each day of the 2.5 times longer duration, which may not be the correct interpretation.

Thank you for catching these potentially misleading formulations. We adapted the wording of the respective sentences:

On lines 17-18 (original draft) we changed "While cyclones generally decrease SIC compared to non-cyclone conditions in cold and warm season, the impact of cyclone clusters is approximately two times stronger and lasts 2.5 times longer than for solitary cyclones" to "While cyclones generally decrease SIC compared to non-cyclone conditions, cyclone clusters impact the ice approximately 2.5 times longer and cause two times stronger overall SIC-reductions than solitary cyclones"

On line 112 (original draft) we changed "the cyclone cluster impact on SIC is 2.3 times stronger and lasts 2.6 times longer than for solitary cyclones" to "the passage of cyclone clusters lasts 2.6 times longer than for solitary cyclones and the resulting overall SIC reduction is 2.1 times stronger" The change from "2.3" to "2.1" is due to the adapted domain and time period definition (see previous comments).

On lines 119-120 (original draft) we changed "the magnitude of the cyclone cluster impact is 1.6-1.8 times stronger and it lasts 2.6 times longer than for solitary cyclones" to "the magnitude of the cyclone cluster impact is 1.2-1.7 times stronger and their passage lasts 2.3-2.6 times longer than for solitary cyclones" Again, changes in the specific numbers are due to the adapted time periods and domains.

On lines 259-260 (original draft) we changed "A key result is that during their passage, clusters of two or more cyclones cause a SIC-reduction (relative to non-cyclone conditions), which is approximately twice as strong and lasts 2.5 times as long as for solitary cyclones (Table 1)" to "A key result is that

clusters of two or more cyclones impact the ice approximately 2.5 times longer and cause two times stronger overall SIC-reductions than solitary cyclones (Table 1)"

19: If there's room in the abstract, it might be nice to highlight that these intensified SIC losses occur even within fixed SIC categories.

Thank you for this suggestion, we added this information at lines 18-19: "The amount of SIC-loss due to cyclone clusters scales with the intensity and number of clustered storms, and intensified SIC-loss occurs from 2004-2024 compared to 1979-1999, even within fixed SIC-categories."

75, 84: This might be somewhat superfluous because I recognize the differences are quite small, but could a third row be added to Supplementary Figs. 1 and 2 showing the differences from Fig. 1 to make the comparisons a bit easier?

Thank you for this suggestion, we followed your advice and included additional panels to Supplementary Figs. 1 and 2 indicating the difference from Fig. 1. We further included a significance test for the differences in the clustering rate (student's t-test on 95% confidence level). The wording at lines 76 and 85 has been changed to:

Line 76: "Lowering the time threshold from 48 to 36 hours reduces the number of detected clusters, particularly outside the Arctic along the cold-season storm tracks, but does not qualitatively alter the results (Supplementary Fig. 1)."

Line 87: "Throughout basically the entire study domain, seasonal clustering rates did not change significantly between the periods 1979-1999 and 2004-2024 (Supplementary Fig. 2)."

118: I'm intrigued by the regions of positive SIC change in Fig. 2d in the Chukchi Sea and by the Canadian archipelago. Do you have any insight to why clusters would have a less negative ice impact than solitary cyclones in those regions?

Thanks for noticing these interesting features. The patches of positive SIC changes in Fig. 2d are mostly found in coastal regions (of the Chukchi Sea and the Canadian archipelago as well as in the Laptev and Kara Seas). Thus, a possible explanation could be that cyclone clusters advect sea ice towards the coastline, where it accumulates. If the affected near-coast grid-cells initially have SIC values below 100% - as may occur during the warm season - this accumulation can lead to increase in SIC. Similar features are not found in the cold season, when the sea-ice cover in these regions is typically compact. Under such conditions, sea-ice advection towards the coast would be expected to increase the ice thickness rather than ice concentration. Many of the grid-cells exhibiting positive SIC changes in Fig. 2d also show positive SIC responses to solitary cyclones (Fig. 2e). However, these responses are much weaker and do not reach statistical significance. This suggests that the underlying mechanism could be the same in both cases, but that its effect is substantially stronger during cyclone clusters.

Fig. 5: This is an interesting figure! I'm finding it a little difficult to discern the colors of the boxes (particularly near the center of the scale and for the pack ice categories) given all the additional information on the plot. Perhaps the values of the colors could be labeled along the x-axis or at the top of the whiskers.

Thank you pointing out this weakness in the figure design. As you point out in your comment, the figure already contains a lot of information. Thus, we would prefer not to add even more content (numbers in addition to the box colors). However, we changed the colormap of the box colors to include only a few discrete levels instead of being continuous, so that the different box-colors can be better distinguished (see excerpt from new Figure 5 below):

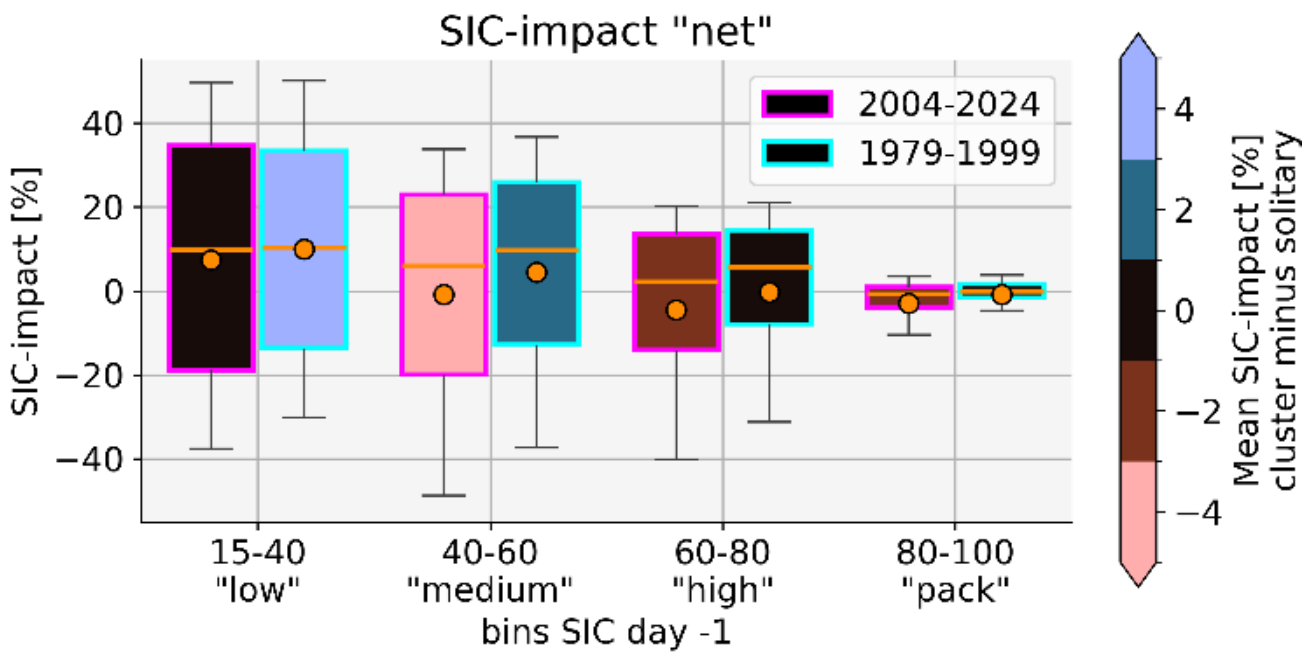

Supplementary Figs. 4, 6: Could the respective Eurasian and Amerasian domains be added for easier comparison?

In response to one of the other reviewer comments, we decided to remove Supplementary Figs. 4 and 6 from the Supplement.

316-318: Is April excluded for a similar reason?

Indeed, April is excluded for similar reasons, namely because in contrast to the following months, no clear negative SIC-change for non-cyclone conditions is found, specifically in the Atlantic marginal seas of the Arctic (see excerpt from Figure S7 below). As described at lines 328-330 of the revised manuscript, we decided to group those months where the sea-ice concentration in the Arctic is usually (in the absence of cyclones) increasing (cold season) or decreasing (warm season), to facilitate the interpretation of the cyclone anomalies in SIC changes.

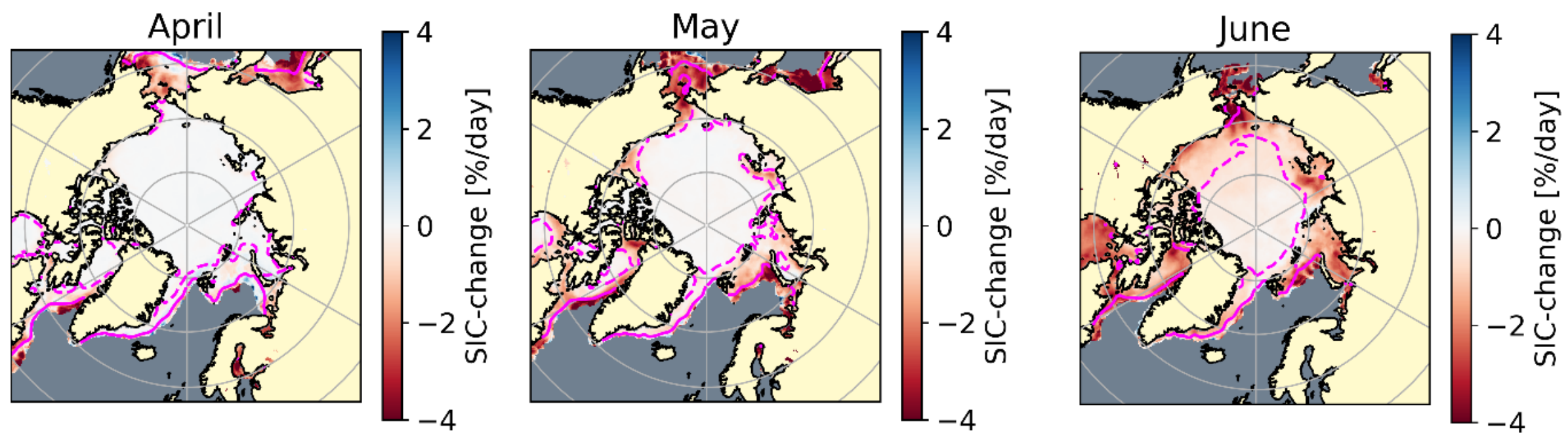

Fig. S7. Non-cyclone reference SIC-change. Non-cyclone reference SIC-change for each month of the years 2004-2024. Solid (dashed) magenta line indicates 15% (90%) mean SIC contour.

346: Please clarify over what time interval the non-cyclone reference period is averaged over. Are separate references used for the 1979-1999 and 2000-2024 time periods?

Thank you for making us aware of this missing information. The non-cyclone reference is indeed averaged separately for the Old and New Arctic period. We added this information at line 370 of the revised manuscript:

“The non-cyclone reference is calculated separately for each calendar month and for the periods 1979-1999 and 2004-2024. Each cyclone event is then compared against the corresponding monthly reference.”

Response to REVIEWERS' COMMENTS (NCOMMS-26-009040A)

We would like to thank the reviewers for the helpful feedback on our manuscript. Our responses can be found below in blue color.

Reviewer #1 (Remarks to the Author):

The authors have adequately addressed my comments and revised the manuscript accordingly. The manuscript is now suitable for publication.

Thank you for this positive feedback and the helpful comments in the previous round of revision.

Reviewer #2 (Remarks to the Author):

Review of NCOMMS-26-00940A

Title: On the relevance of serial cyclone clustering for Arctic sea ice

Author(s): Lars Aue, Sofie Tiedeck, Peter Finocchio, Timo Vihma, Petteri Uotila, Gunnar Spreen, and Annette Rinke

Summary and Key Results: This manuscript aims to quantify the impact of cyclone clusters on very rapid sea ice loss events in the Arctic. Cyclone clusters occur when there are several cyclones that follow in near spatial and temporal proximity to each other. The analysis is conducted using ERA5 atmospheric reanalysis, ERA5 sea ice concentration (SIC), and a freely available cyclone tracking algorithm. The authors find that cyclone clusters contribute to significantly more loss in SIC over a longer amount of time compared to single (solitary) cyclone events. They also find that the losses in SIC have increased in recent times.

I thank the authors for carefully and adequately considering my comments on the previous version. I think this article is nearly ready for publication and will make an excellent contribution to Nature Communications.

Thank you for this positive feedback. We adapted the manuscript according to the suggested minor comments below.

Minor edits:
1) Figure 4 caption: indicate share → indicate the share
Thank you for capturing this. We changed the wording in the caption of Figure 4 accordingly.
2) 337: pronounced stronger → more strongly pronounced
Thank you for capturing this. We changed the wording at line 337 accordingly.
3) 387, 388: Comma after equation, no indent before where on following line
Thank you for capturing this. We changed the formatting at lines 387,388 accordingly.

Reviewer #3 (Remarks to the Author):

I appreciate the authors' thorough responses and effort in improving and clarifying the manuscript. My concerns have been addressed, and I have no further comments.

Thank you for this positive feedback and the helpful comments in the previous round of revision.

Reviewer #3 (Remarks on code availability):

The code appears well documented and the Readme file is clearly written. I appreciate the use of a demo case, which ran successfully.

Thank you for investing the time to review our code and test the demo case. We are glad to hear that it ran successfully.

Review of NCOMMS-26-00940

Title: On the relevance of serial cyclone clustering for Arctic sea ice

Author(s): Lars Aue, Sofie Tiedeck, Peter Finocchio, Timo Vihma, Petteri Uotila, Gunnar Spreen, and Annette Rinke

Summary and Key Results: This manuscript aims to quantify the impact of cyclone clusters to very rapid sea ice loss events in the Arctic. Cyclone clusters are when there are several cyclones that follow in near spatial and temporal proximity to each other. The analysis is conducted using ERA5 atmospheric reanalysis, ERA5 sea ice concentration (SIC), and a freely available cyclone tracking algorithm. The authors find that cyclone clusters contribute to significantly more loss in SIC over a longer amount of time compared to single (solitary) cyclone events. They also find that the losses in SIC have increased in recent times.

Overall, this manuscript provides a convincing case that cyclone clusters have a larger impact on SIC decreases than single cyclones. My expertise lies mostly within atmospheric dynamics, yet I struggled to understand the section "Role of initial SIC prior to cyclone passage." In particular, I find the last part of the analysis confusing (the decomposition of the changed SIC impact), and it is not clear what it ends up adding to the conclusions. Thus the presentation of this section could need additional work, given that Nature Communications aims to reach a broad, global audience of professional researchers, academics, and specialists across all natural sciences. There are some other issues that need addessing, particularly in the methods, with some other issues in clarity needed. Once these issues have been addressed, I think this article will make a excellent contribution to Nature Communications.

Significance: It is reasonable to expect that a series of cyclones will lead to a larger net reduction of sea ice than single cyclone events. This study quantifies this expectation, and furthermore shows that these impacts are increasing in the New Arctic. However, I think the societal relevance could be better stressed. For example, cyclones, reductions in sea ice, thawing permafrost, and increasing river flows are leading to notable increases in hazardous weather events in and around the Arctic region (e.g., Valkonen et al. 2021; Zhang et al. 2023). Coastal regions are now facing substantial erosion and flooding, particularly when impacted by cyclones. Away from coastal regions, reduced sea ice increasingly reveals oil reserves within the Arctic Ocean, motivating bordering countries to extend their sovereignty claims in international waters (e.g. Gerhardt et al. 2010; Carlson et al. 2013) and has led to a dramatic increase in shipping in the Arctic due to the increased accessibility and shorter shipping routes, particularly during the summer (e.g., Stephenson et al. 2013; Stephenson and Smith 2015).

Data and methodology, analytic approach, and suggested improvements:

1) PIOMAS sea ice thickness is used in the sea ice thickness analysis, but it is not mentioned or described in the data and methods section.

2) The authors separate data into two periods: one corresponding to the so-called "Old Arctic" (1979–1999) and the other the "New Arctic" (2000–2024). This idea is reasonable, except I wonder why the two periods don't have the same number of years. This makes it harder to compare because the "New Arctic" has a much larger sample size. Seems like it would be most simple and fair to just have the "New Arctic" start at 2004. This would likely make the authors' results stronger, since 2007 was the first drastic reduction in year-to-year sea ice extent.

3) The analysis seems incomplete when choosing not to include April, September, and October. The text does not provide very clear reasons as to how the authors decided how to exclude certain months. For example, they state the October is excluded because it "differs substantially" from the following months. But what does "substantial" mean (line 318)? Is there a number being used to support this conclusion? I think the authors are ultimately looking to divide the sample into two periods within each year: One where sea ice is thicker and extensive with relatively low spatial variability (limited mainly to the MIZs), and the other where sea ice is thinner and less extensive with relatively high spatial variability. Perhaps the combination of these specific factors is quantifiable. Or if the authors expect a different relationship between cyclones and sea ice, a "shoulder seasons" analysis could also be included.

4) 83–84: What would make clustering rates be considered "similar" vs. being considered "not similar?" Is there a threshold? Or is it arbitrary?

5) 114: The word "generally" is not needed. The comparison is between -0.3% and -0.4%, which I doubt is quantfiably different.

Clarity and Context:

1) How are SLP "anomalies" defined (line 324)? Is it the anomaly with respect to a long-term climatology, or is it a spatial anomaly? If it is respect to a long-term climatology, what years are used? And is it daily, monthly, or seasonal averages that are being subtracted?

2) What exactly is "cluster intensity?" Cyclone intensity is described on lines 332–334, but it's not obvious to me exactly how this applies to a cluster of cyclones.

3) For both the Eurasian cold season (Figure 5) and Amerasian warm season (Figure 6) analyses, the authors perfrom a hypothesis test that changes in cyclone cluster trajectories between the "new" and "old" Arctic periods could be responsible for dynamically-driven SIC loss. Some more context is needed for how this test is expected to eliminate or support the possibility that the SIC losses are dynamically driven. In both cases, there is no statistical significance (though the authors explcitly point this out in the text for the former but not the latter). Thus the authors seem to be eliminating the possibility of dynamically-driven changes in SIC since there is no statistical significance. They then show evidence that SIT is decreasing between the two periods. But this would be dynamically-driven also (i.e., storms can more easily impact sea ice if the ice is thinner). Is this what the authors are trying to show? If so, it needs to be better explained. If not, perhaps this analysis is not necessary and could be removed or strongly condensed.

4) On lines 233–235, "all samples" should be changed to "all samples where there is SIC>15%."

5) While ice-free summers will limit the impact that warm-season cyclones can have on sea ice in the future, won't this just shift the impacts into the shoulder seasons (lines 286–288)? There is no reason to expect anything to be different about the causes.

Literature Review:

1) 28: To be consistent with Cavallo et al. (2025), the sea ice loss is not just extreme, but it is also very rapid (i.e., VRILEs).

2) The literature review considers "summer" and "winter" cyclones, but the analysis is for "warm" and "cold" season cyclones. Are the two meant to be equivalent? And what about the shoulder seasons? Or are the authors trying to divide the analysis into "warm" season and "non-warm" season?

Typos and misspellings:

1) 17: season → seasons

2) 309: 25km → 25 km

3) 361–362: There should be a comma at the end of the equation labeled as equation (2), and "where" should not be indented on line 362.

References

Carlson, J. D., C. Hubach, J. Long, K. Minteer, and S. Young, 2013: Scramble for the Arctic: Layered Sovereignty, UNCLOS, and Competing Maritime Territorial Claims. *SAIS Review of International Affairs*, **33 (2)**, 21–43.

Cavallo, S. M., M. C. Frank, and C. M. Bitz, 2025: Sea ice loss in association with Arctic cyclones. *Communications Earth & Environment*, **6 (1)**, 1–9, doi:10.1038/s43247-025-02022-9.

Gerhardt, H., P. E. Steinberg, J. Tasch, S. J. Fabiano, and R. Shields, 2010: Contested sovereignty in a changing Arctic. *Annals of the Association of American Geographers*, **100 (4)**, 992–1002.

Stephenson, S. R. and L. C. Smith, 2015: Influence of climate model variability on projected Arctic shipping futures. *Earth's Future*, **3 (11)**, 331–343.

Stephenson, S. R., L. C. Smith, L. W. Brigham, and J. A. Agnew, 2013: Projected 21st-century changes to Arctic marine access. *Climatic Change*, **118 (3-4)**, 885–899.

Valkonen, E., J. Cassano, and E. Cassano, 2021: Arctic cyclones and their interactions with the declining sea ice: A recent climatology. *Journal of Geophysical Research: Atmospheres*, **126 (12)**, e2020JD034 366, doi:10.1029/2020JD034366.

Zhang, X., H. Tang, J. Zhang, J. E. Walsh, E. L. Roesler, B. Hillman, T. J. Ballinger, and W. Weijer, 2023: Arctic cyclones have become more intense and longer-lived over the past seven decades. *Communications Earth & Environment*, **4 (1)**, 348.
